# A Physiologically-Motivated Compartment-Based Model of the Effect of Inhaled Hypertonic Saline on Mucociliary Clearance and Liquid Transport in Cystic Fibrosis

**DOI:** 10.1371/journal.pone.0111972

**Published:** 2014-11-10

**Authors:** Matthew R. Markovetz, Timothy E. Corcoran, Landon W. Locke, Michael M. Myerburg, Joseph M. Pilewski, Robert S. Parker

**Affiliations:** 1 Department of Chemical and Petroleum Engineering, Swanson School of Engineering, University of Pittsburgh, Pittsburgh, PA, United States of America; 2 Pulmonary Allergy and Critical Care Medicine, University of Pittsburgh, Pittsburgh, PA, United States of America; 3 McGowan Institute of Regenerative Medicine, University of Pittsburgh, Pittsburgh, PA, United States of America; 4 Clinical Research, Investigation, and Systems Modeling of Acute Illness (CRISMA) Laboratories, University of Pittsburgh, Pittsburgh, PA, United States of America; 5 University of Pittsburgh Cancer Institute, University of Pittsburgh, Pittsburgh, PA, United States of America; 6 Department of Bioengineering, Swanson School of Engineering, University of Pittsburgh, Pittsburgh, PA, United States of America; The Hospital for Sick Children and The University of Toronto, Canada

## Abstract

**Background:**

Cystic Fibrosis (CF) lung disease is characterized by liquid hyperabsorption, airway surface dehydration, and impaired mucociliary clearance (MCC). Herein, we present a compartment-based mathematical model of the airway that extends the resolution of functional imaging data.

**Methods:**

Using functional imaging data to inform our model, we developed a system of mechanism-motivated ordinary differential equations to describe the mucociliary clearance and absorption of aerosolized radiolabeled particle and small molecules probes from human subjects with and without CF. We also utilized a novel imaging metric *in vitro* to gauge the fraction of airway epithelial cells that have functional ciliary activity.

**Results:**

This model, and its incorporated kinetic rate parameters, captures the MCC and liquid dynamics of the hyperabsorptive state in CF airways and the mitigation of that state by hypertonic saline treatment.

**Conclusions:**

We postulate, based on the model structure and its ability to capture clinical patient data, that patients with CF have regions of airway with diminished MCC function that can be recruited with hypertonic saline treatment. In so doing, this model structure not only makes a case for durable osmotic agents used in lung-region specific treatments, but also may provide a possible clinical endpoint, the fraction of functional ciliated airway.

## Introduction

Cystic Fibrosis (CF) is an autosomal recessive disease that arises from a defect in the Cystic Fibrosis Transmembrane Conductance Regulator (CFTR) gene. CF affects multiple organ systems, with the most detrimental effects occurring in the lungs [1]. CFTR encodes an anion channel on epithelial surfaces, that is dysfunctional or absent from the CF epithelium. The associated loss of 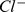 and 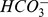 secretion along with a tendency to hyperabsorb 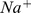 through the epithelial sodium channel (ENaC) results in osmotic gradients that favor rapid absorption of the airway surface liquid (ASL) layer, leading to dehydrated airway mucus secretions and impaired mucociliary clearance (MCC) [2], [3]. The inability to clear pathogens via MCC leads to chronic infection, inflammation, airway damage, and premature respiratory failure.

Inhaled agents that reverse osmotic gradients in the airways are used to treat the ASL dehydration defect associated with CF. Hypertonic saline (HS) is one such osmotic agent that has been shown to increase both MCC and lung function in patients with CF [4], [5]. More recently CFTR modulators have been developed that substantially improve lung function in individuals with specific CFTR mutations [6].

**Figure 1:**
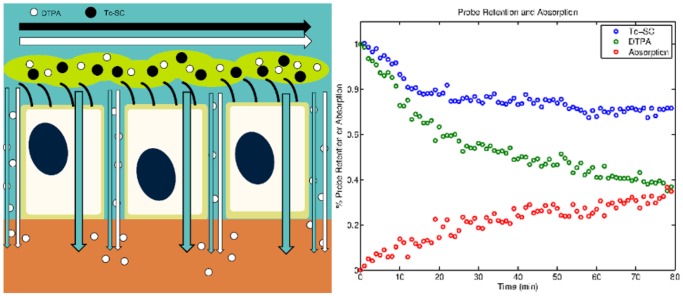
Left panel: schematic of transport routes in the airway epithelium. The black and white horizontal arrows represent mucociliary clearance of Tc-SC and DTPA, respectively. DTPA (downward white arrow) absorbs across the airway epithelium via the paracellular route. Water (downward blue arrow) also absorbs across the epithelium via the transcellular and paracellular route. Right panel: normalized Tc-SC (blue circles) and DTPA (green circles) retention curves with the difference between the respective datasets at each time point shown as absorption (red circles) in CF subject 8.

Outcome measures and biomarkers that quantify the basic pathophysiology of CF lung disease are needed to allow for the rapid screening of new therapeutics for CF. Ideally, these screening methods seek to quantify the basic pathophysiology of CF lung disease. Mucociliary clearance scans, which quantify the clearance of a radiolabeled particulate from the lungs, are one such functional imaging method for studying outcomes in CF. We have expanded this method to include measuring the clearance of a radiolabeled small-molecule that can be absorbed as well as cleared via MCC. Similar techniques have been used in the past to resolve the individual components of lung clearance [7], [8]. Our *in vivo* method utilizes Technetium 99m sulfur colloid (Tc-SC) as the non-absorbable particle probe and Indium 111-DTPA (DTPA) as the small molecule probe. The probes are delivered together in a liquid aerosol. Figure 1 presents a schematic for pharmaceutical clearance from the airway epithelium. We assume that MCC clears the probes at similar rates and that the difference in their clearance rates is therefore associated with the absorption of the small molecule. Previous *in vitro* studies have demonstrated a relationship between DTPA and airway surface liquid absorption rates [9]. DTPA absorption is increased both *in vivo* in CF airways and *in vitro* in CF airway cell cultures [9], [10]. Decreases in DTPA absorption after osmotic therapies have also been demonstrated *in vitro* and *in vivo* [9], [10]. Thus, multi-probe methods provide measurements of both MCC and ASL absorption rates [10]. Successful CF therapies should restore ASL volume by correcting defective airway epithelial ion transport [11], [12]. The restoration of airway hydration should be rapidly detectable through these multi-probe imaging methods as decreased liquid absorption rates are reflected through slower absorption of DTPA and improvements in MCC cause increased Tc-SC clearance.

Herein we present a compartment-based model of particle and small molecule clearance from the human respiratory tract that uses functional imaging data to inform model structure and quantitate parameter values. Our goal in developing this model is to better resolve the specific physiological mechanisms that contribute to the composite functional imaging result. We believe that these mechanism-specific measurements may provide more sensitive and specific evaluations of therapeutic efficacy than the currently used image-derived metrics. The challenge is to construct a mathematical representation of the physiology that can resolve the underlying mechanisms and their interactions.

There have been a number of compartment-based pharmacokinetic (PK) models for inhaled pharmaceuticals [13], [14], [15], [16], [17], but to our knowledge none have considered the PK behavior of the radiopharmaceuticals used for functional imaging from which our data originates, nor has anyone assessed absorption kinetics with the methodology we have employed. We hypothesize that by dividing the lung into a solely absorptive peripheral lung region and separate central lung region that has both absorptive and MCC capability, and by subdividing this central lung region into fractions with, and without, functional ciliated airway, we can reproduce the dynamics of simultaneous particle and small molecule probe clearance from the lung. We further hypothesize that (i) functional ciliated airway clears at the same rate in patients with CF and non-CF subjects; (ii) the fraction of functional ciliated airway (FFCA) is decreased in CF; and (iii) FFCA can be increased through inhalation of HS. Our models provide estimates of FFCA, MCC, and large airway and peripheral lung absorption, the latter of which we believe is related to liquid hyper-absorption in CF small airways. These measured parameters may substantially expand the utility of our functional imaging measurement by providing more mechanistic insight into lung physiology and response to therapeutic agents (a list of all abbreviations and their definition used in this work can be found in Table 1).

**Table 1:** Abbreviations and constants in the work presented herein.

Abbreviation	Description
AIC	Akaike Information Criterion; a Metric of Model Appropriateness
ASL	Airway Surface Liquid Layer
C	Imaging Region of Interest About the Central Lung; Analogous to 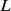 in Model
CF	Cystic Fibrosis
CFTR	Cystic Fibrosis Transmembrane Conductance Regulator
CI	Confidence Interval
DMEM	Dulbecco's Modified Eagle Medium
DTPA	Indium-111 Diethylene Triamine Pentaacetic Acid
ENaC	Epithelial Sodium Channel
FFCA	Fraction of Functional Ciliated Airway
HBE	Human Bronchial Epithelial (cell)
HS	Hypertonic Saline
IS	Isotonic Saline
k	Number of Free Parameters in Model
MCC	Mucociliary Clearance
N	Number of Data Points Analyzed for Model Fit
ODE	Ordinary Differential Equation
PK	Pharmacokinetic
Tc-SC	Technetium-99m Sulfur Colloid
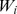	Weighting Factor of the 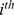 Element of the Dataset for AIC
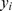	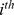 Value of Dataset
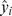	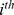 Value of Model Fit

## Results

### Model Synthesis and Assumptions

**Figure 2:**
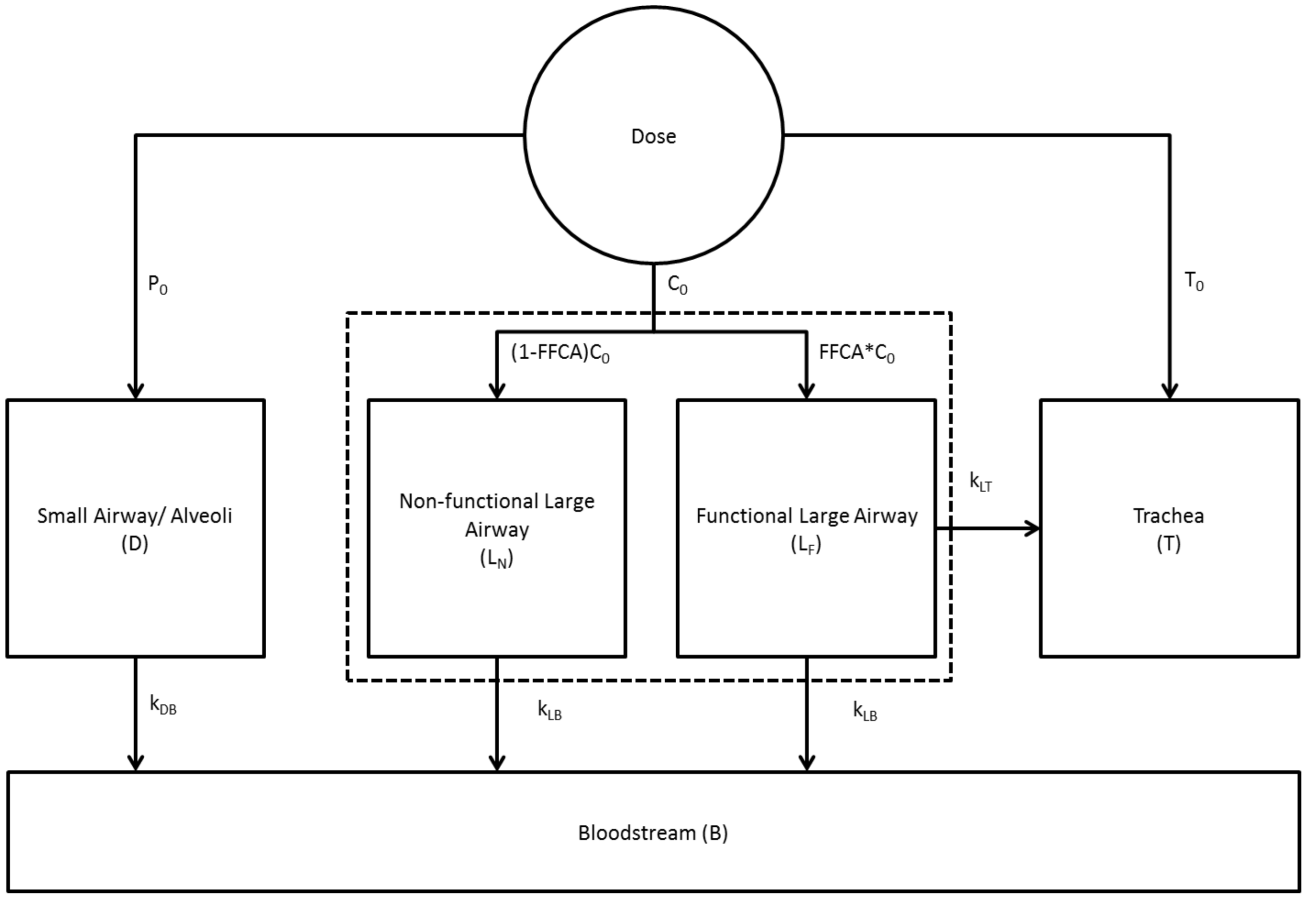
Schematic model structure describing the clearance of small molecule and particle probes from the lung. Initial depositions: 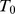 = trachea, 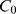 = central, 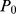 = peripheral. See Table 1 for full list of model abbreviations.

Starting from the original model of Sakagami [15], we extend it structurally to account for the dynamics and kinetics observed in the clinical data set. Founded on a basic understanding of lung anatomy and physiology, the lung structure can be lumped into two key dynamical structures as shown in Figure 2. Additional assumptions made in synthesizing this model are as follows:

- A fraction of the large airways (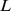), which are represented in the central (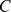) lung region of interest (ROI) [18], has functional MCC, with MCC in the remainder of 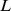 being non-functional. This allows the subdivision of 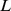 into two sub-compartments: 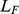, which has functional MCC, and 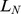, which has no functional MCC.
- The initial doses of the radiopharmaceuticals delivered to either the central (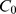) or peripheral (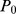) lung regions are described as the number of counts within 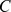 or in the area outside of 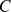 but within the whole lung ROI in the first image, respectively [10] (Figure S1 provides a visual representation of the C and P ROIs).
- 10% of inhaled radiopharmaceuticals were assumed to deposit in the trachea (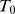) [15]. However, neither counts in 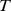 nor the bloodstream (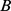) have a unique impact on the clearance of the Tc-SC and DTPA.
- MCC in the distal lung 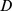, which contains small and intermediate-sized airways in addition to alveoli [18], is a slow process; given the 80-min timescale of our data this rate cannot be resolved with confidence. As a result, the MCC term from D to L, 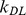, is neglected.

The result of these assumptions is a three-compartment model for MCC in the lung wherein two compartments (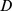 and 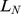) are absorptive and one compartment (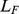) has MCC in addition to absorptive clearance, as shown in Figure 2.

### Model Equations

Equations modeling the transport of both of the probes are outlined below. The model parameters 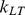, 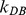, 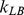 and FFCA, the fraction of 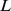 with functional MCC, were determined by nonlinear least-squares regression as described in **Methods**. For Tc-SC clearance, 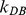 and 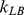 are set equal to 0. The mathematical description of this model, written as ODEs representing the change in counts in each model compartment, is as follows (see Table 2 for full list of model abbreviations): 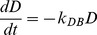
(1)

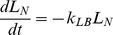
(2)

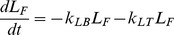
(3)

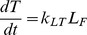
(4)

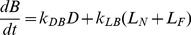
(5)


**Table 2:** Model parameters in the work presented herein.

Parameter	Description
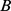	Bloodstream Model Compartment (units counts)
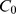	Initial Counts in 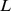 (units counts)
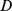	Distal Lung Model Compartment (units counts)
FFCA	Free Parameter; Fraction of 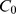 in 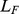 (units counts)
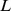	Large Airway Model Super-Compartment (units counts)
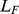	Sub-compartment of 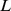 with Functional MCC (units counts)
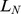	Sub-compartment of 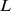 without Functional MCC (units counts)
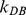	Free Parameter; Rate Constant of Absorption in 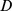 (units 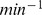 )
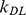	Free Parameter (Unused in Final Model); Rate Constant of MCC from 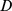 to 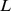 (units 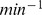 )
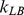	Free Parameter; Rate Constant of Absorption in 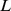 (units 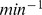 )
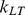	Free Parameter; Rate Constant of MCC from 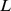 to 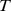 (units 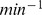 )
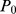	Initial Counts in 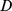 (units counts)
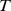	Trachea Model Compartment (units counts)

### Model Fit and Predictions

**Figure 3:**
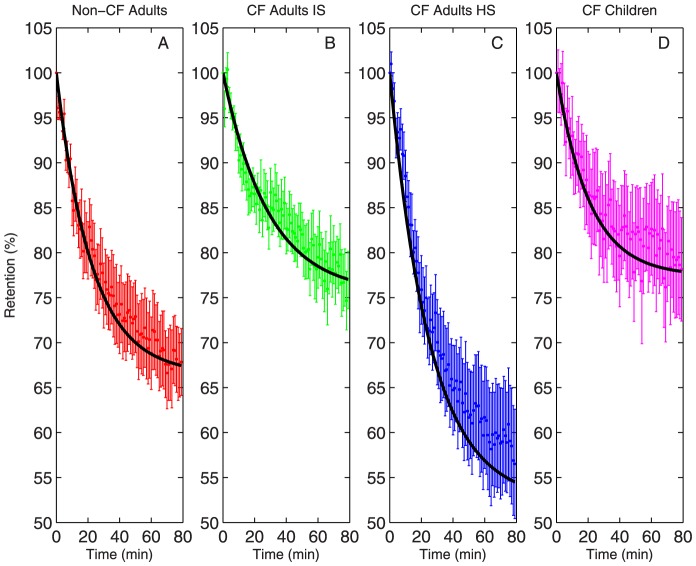
Tc-SC retention (mean 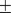
SEM) vs. model fit (black line) for four patient subgroups. (A) Non-CF subjects (n = 9) (B) patients with CF (n = 12). (C) patients with CF after inhalation of hypertonic saline (n = 11). (D) Pediatric patients with CF. Data from [10].

The ability of our model to reproduce therapeutic response after the inhalation of hypertonic saline was studied using imaging data from patients with CF who inhaled isotonic saline (IS) and hypertonic saline on alternating study days [10]. Retention of Tc-SC, which is a metric of MCC, is shown with model fit for each patient group in Figure 3 [3]. Data from pediatric patients with CF and healthy controls who inhaled isotonic saline is also shown. The model captured the dynamics of MCC in all patient groups. The previously described increases in MCC associated with HS inhalation are apparent in the image-derived data and the model fits. However, as previously reported, no statistical differences in baseline MCC were detected between the CF IS, pediatric CF, and control groups [10].

**Figure 4:**
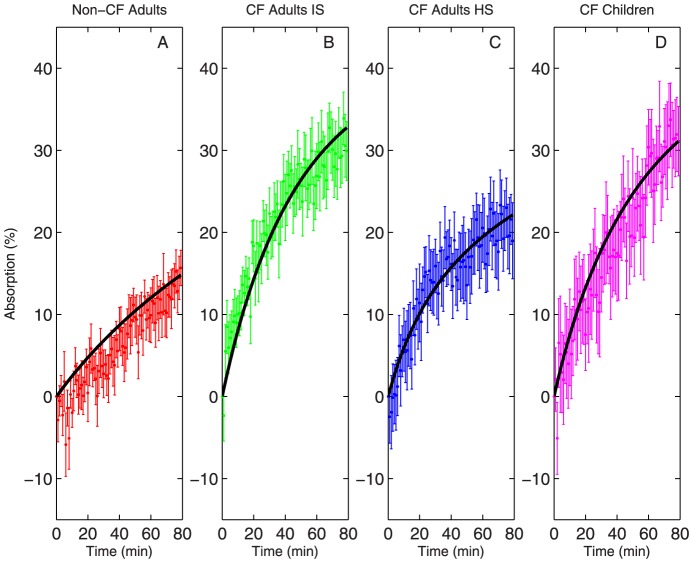
DTPA absorption (mean 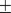
SEM) vs. model fit (line) for four patient subgroups. (A) Non-CF subjects (n = 9) (B) patients with CF (n = 12). (C) patients with CF after inhalation of hypertonic saline (n = 11). (D) Pediatric patients with CF. Data from [10].

DTPA is assumed to deposit in the same regions and clear by MCC at the same rate as Tc-SC; DTPA absorption is then captured by regressing the parameters 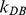 and 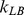. Additionally, our previous studies have shown that the absorption of DTPA indicates changes in the absorption of ASL [9]. As such, the effect of hypertonic saline on liquid absorption, using DTPA as an analog, is shown in Figure 4, along with the model fit for each group [10]. The model again captured the dynamics of absorption in all groups. Increased baseline rates of DTPA absorption are apparent in both adult and pediatric CF groups, as previously described [10].

**Figure 5:**
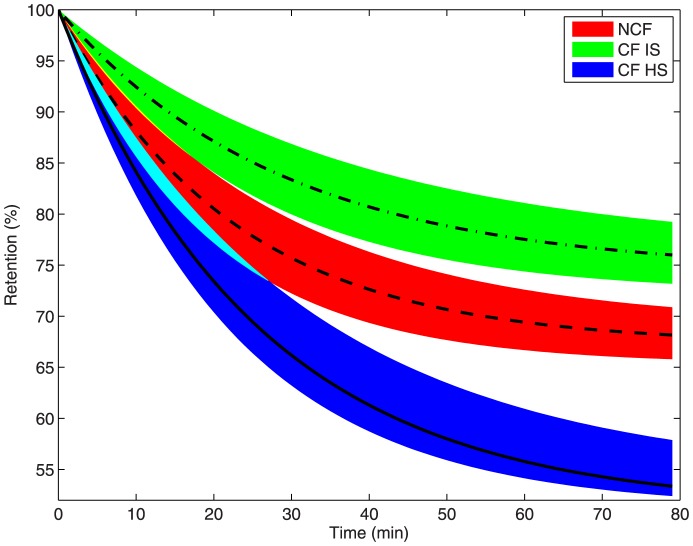
Estimated Tc-SC retention trajectories for three patient subgroups. Non-CF (red with dashed line), CF IS (green with dotted dashed line), CF HS (blue with line). Shaded envelope characterizes 95% confidence interval (CI) of retention prediction by the model. Pediatric CF subjects not shown due to overlap with adult CF.

Our model predicts that both Tc-SC retention and liquid absorption will follow trajectories within narrow envelopes based on the predicted upper and lower 95% confidence intervals (CIs) of the model parameters. These CI envelopes are derived using all possible combinations of the upper and lower 95% CIs and nominal values of the model parameters to generate the extreme values of model predictions. Figure 5 shows that predicted model trajectories lead to different final values of Tc-SC retention for all adult patient groups, with no overlap in trajectory envelopes of CF IS and non-CF groups for t>18 min and no overlap between non-CF and HS trajectories for t>27 min. In other words, the three adult patient subgroups have characteristically different total MCC behavior. Pediatric CF clearance trajectories closely resemble those of adult patients with CF, but are not shown for ease of interpretation. Patients with CF display less MCC than non-CF subjects, which appears to contradict our finding in [10]. However, the present work evaluates the entire time course of clearance, while the end of study value (t = 80 min) was used as the evaluation time point in [10]. By using the entire data sequence, particularly the dynamic response at short times, we are able to parametrically estimate and differentiate - with confidence - the MCC and absorption dynamics between patients with CF and non-CF subjects. These analyses also indicate that MCC function in patients with CF can be rescued by HS treatment, which caused total MCC to exceed that of non-CF subjects.

**Figure 6:**
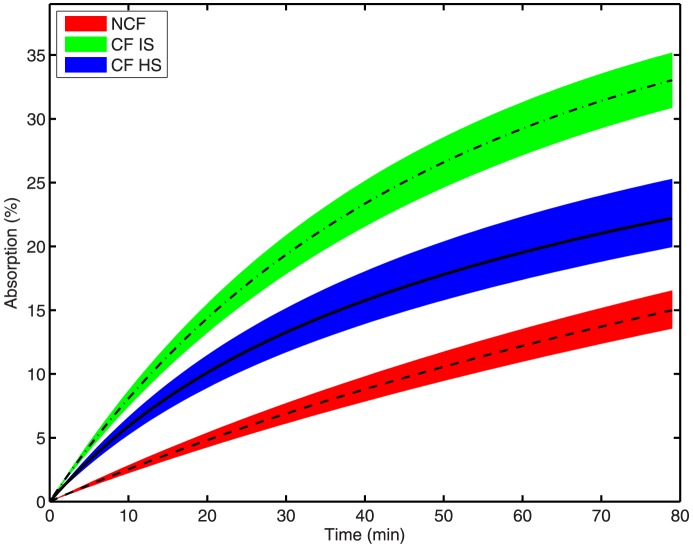
Estimated DTPA absorption trajectories for three groups. Non-CF (red with dashed line), CF IS (green with dotted dashed line), CF HS (blue with line). Shaded envelope characterizes 95% confidence interval (CI) of retention prediction by the model. Pediatric CF subjects not shown due to overlap with adult CF.

Figure 6 shows that the model-predicted trajectories for liquid absorption have no overlap for all t>0. Pediatric CF trajectories again resemble those of adult patients with CF, but are not shown. Patients with CF have higher absorption than non-CF subjects, which can be partially remediated by HS treatment. Combined, the results in Figures 5 and 6 indicate that HS has two distinct mechanisms of action, both rescuing MCC and ameliorating liquid hyperabsorption [4].

**Figure 7:**
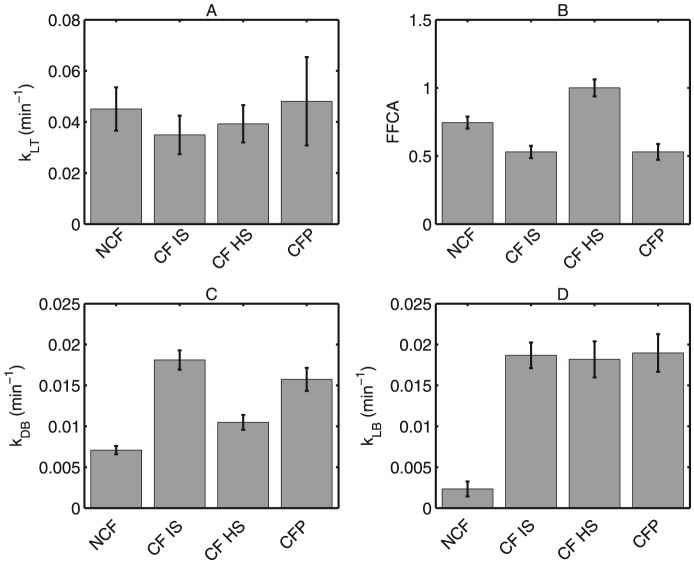
Least-squares solutions for model parameters are shown with 95% confidence intervals for four groups: Non-CF (NCF), CF HS, CF IS, and Pediatric CF (CFP). (A) MCC rate constant (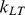) (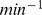). (B) Fraction (FFCA) of large airway with functional MCC. (C) Peripheral lung absorptive rate constant (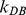) (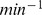). (D) Large airway absorptive rate constant (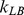) (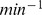).

It has been proposed that the apparent increase of MCC following HS treatment occurs by increasing the rate at which MCC occurs [4], [19]. Our model suggests that the intrinsic rate of MCC is the same in all patient groups, and the rescue of MCC can be attributed to the recruitment of inactive portions of the airway. Figure 7A shows that the rate of MCC, 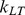, is not significantly different between all patient groups (p>0.05). However, the parameter FFCA (Figure 7B), which describes the fractional area of large airways with functional MCC, is significantly lower in patients with CF versus non-CF subjects (p<0.05). Patients with CF given HS, however, show a significantly increased FFCA (p<0.05). This would support the concept of increased MCC, not through an intrinsic rate increase, but through an increase in the overall epithelial area contributing to MCC.

Our model also agrees with the previous reports that absorption, represented by 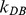 and 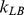 in our model, is increased in patients with CF vs. non-CF subjects (p<0.001 both parameters). The model demonstrates that HS inhalation decreases absorption in the peripheral lung (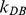), which includes both small and intermediate sized airways and alveolar regions, more than IS inhalation (p<0.001). All model parameters are similar when comparing baseline measurements in the pediatric and adult CF IS groups (p = NS). Our parameters, as well as those of similar studies, are shown in Table 3.

**Table 3:** Pharmacokinetic Lung Clearance Parameter Values.

Study	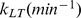	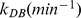
NCF	0.0450 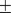 0.0085	0.0071 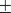 0.0005
CF HS	0.0393 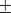 0.0073	0.0105 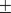 0.0009
CF IS	0.0349 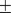 0.0076	0.0181 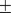 0.0012
CF Pediatric	0.0481 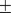 0.0173	0.0157 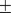 0.0014
Sakagami* [14]	0.022 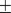 0.002	0.048 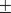 0.003

Values from this study are shown with 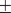
95.

*PK model in isolated perfused rat lung. Three fluorescent markers used, but only the parameter values for sodium fluorescein (F-Na) are shown since 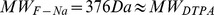.

### *In Vitro* Evaluation of Model Prediction

**Figure 8:**
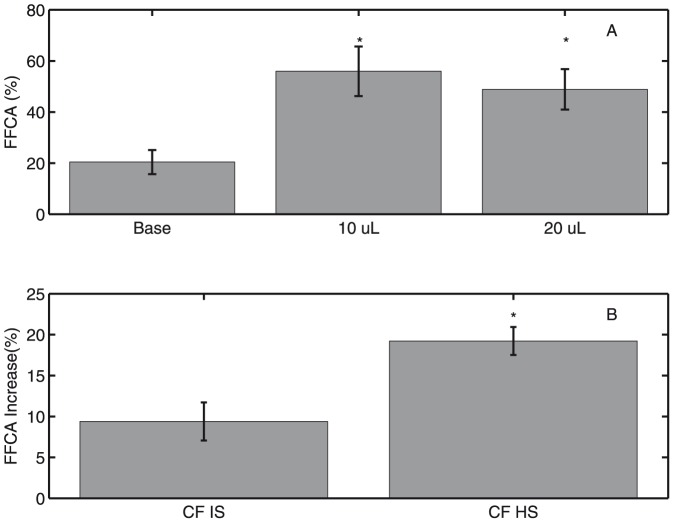
FFCA determined *in vitro* in CF HBE cells is shown 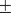 SEM. (A) Activated area is significantly greater between 10 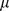
L and baseline (* 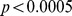) and 2×10 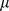
L and baseline (* 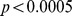). Activated area is not significantly greater between hydrated groups (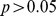). (B) A two-tailed t-test with unequal variances assumed reveals that the increase in active area induced by HS in cells is significantly greater than that of IS (* 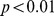).

In order to verify that hydration increases the fraction of functional ciliated area, we imaged CF HBE cultures (6 lines, n = 12) before and after addition of 10 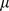
L of Dulbecco's Modified Eagle Media (DMEM) (Sigma-Aldrich, St. Louis, MO, USA), a liquid cell culture media, to the apical surface and again after a second addition of 10 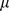
L of DMEM. We determined that hydration of HBE cells by addition of 10 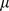
L significantly increases the fraction of functional ciliated area (p<0.0005) above baseline. No further increase in FFCA was observed following a second addition of 10 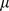
L DMEM (p = NS), as shown in Figure 8A. CF HBE cells were imaged following apical addition of either 5 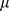
L IS (n = 6) or HS (n = 6). A comparison of relative effect of IS and HS in CF HBE cells is shown in Figure 8B. Both IS and HS additions increase functional area (p<0.05 and p<0.001, respectively). However, HS increases functional area significantly more than IS (p<0.01).

## Discussion

The failure of the CF airway epithelium to secrete 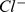 and absorb 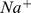 in a normal manner results in a net osmotic gradient that favors rapid absorption of liquid from the ASL, resulting in diminished MCC and an accumulation of dehydrated mucus in the airways. Nuclear imaging methods have been previously developed to measure MCC and have been used as biomarkers of therapeutic efficacy. A novel method that measures both MCC and ASL absorption has been explored more recently [10]. This method involves the inhalation of radiolabeled particle and small molecule probes (Tc-SC and DTPA, respectively). The difference between total DTPA clearance and Tc-SC clearance can be used to estimate the absorption of DTPA, and previous studies have demonstrated: (i) a positive and significant correlation between DTPA and ASL absorption rates [9]; (ii) DTPA absorption response to the use of osmotic therapies both *in vivo* and *in vitro* [10], [9].

We have developed a model that considers the basic mechanisms governing small molecule and particle probe clearance from physiologically distinct compartments of the lung. The combination of these mechanisms is reflected in the imaging result. Compartment or mechanism specific results provide additional insight into CF lung pathophysiology and therapeutic mechanisms of action. They may also provide more sensitive and accurate predictions of therapeutic efficacy than analyses of the composite imaging data as the compartmental description incorporates the inherent time-correlation of mass transport within the model structure.

### Model Structure Selection and Analysis

Our physically descriptive model includes central and peripheral lung compartments. The central zone includes large airways, intermediate and small airways, and alveoli while the peripheral zone includes only intermediate and small airways and alveoli [18], [20] (See Figure S1). The peripheral zone includes both smaller airways and alveoli in a single compartment. The model also includes compartments for the trachea and blood. As determined by Akaike Information Criterion (AIC) [21] analysis, a quantitative metric that weights the model accuracy (as measured by sum-squared error) against the cost of model complexity (quantified by the number of parameters in a model), the most appropriate model given the timeframe of our data assumed zero MCC from the peripheral compartment. Model rate constants describing MCC from the functional large airway compartment (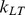), absorption from the small airway/alveolar compartment (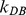), large airway absorption (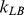), and FFCA were fit to the data. The fixed mathematical structure of the model is able to capture the dynamics of both MCC and the absorption of DTPA in the lungs of all patient groups.

An alternative, and more highly parameterized, model than that shown in Figure 2 would include parameter 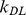 as an MCC rate constant from compartment 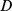 to compartment 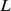, in addition to MCC rate constant 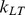 and absorption rate constants 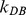 and 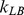. All kinetics were assumed to be first order. Furthermore, the initial dosing of probes was included using a free dosing parameter (C/P), representing the ratio of radiopharmaceutical doses delivered to the large airways and peripheral lung. Under the assumption that Technetium 99m-labeled Sulfur Colloid (Tc-SC) is not absorbed in any lung region, the MCC curve was found to be bi-exponential, as suggested previously [15]. However, regression and analysis of the model parameters via the MATLAB functions *lsqnonlin* (a nonlinear least-squares regression algorithm) and *nlparci* (an algorithm that computes confidence intervals on estimated parameters from the covariance matrix) yielded wide parameter confidence intervals, approximately 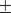
10 times the nominal parameter value. By eliminating 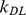 the confidence intervals on parameter estimates are tightened dramatically (the worst case parameter CI from the model herein is 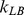 in non-CF subjects which has CI's that are 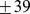
% of the nominal value).

While a two-compartment model, like that proposed by Sakagami in [15], may be used to describe clearance, the parameters may not be able to be estimated with confidence if a physiological rate process captured by a parameter in the model is on the timescale of the experiment, or longer. The 80-minute functional imaging session inherently limits the confidence that can be derived for slower physiological processes. Therefore we hypothesized that the departure from monoexponential clearance seen in the retention curves could be explained by a non-zero steady state value for pharmaceutical retention. While this is aphysiologic over a period of days, an apparent non-zero baseline would manifest if a slow clearance process was occurring and data collection was short by comparison to the half-life of the clearance. This behavior can be realized in a number of ways in a kinetic model. In the present study, it is most easily incorporated by adding a new compartment that has no apparent MCC mechanism over the experimental timescale. Thus, while we believe that MCC in D can be observed on a longer timescale, we consider its mechanism to be practically unidentifiable (i.e. there is insufficient data to inform this parameter) over the timescale examined in the present work [22].

The model structure presented in Figure 2, and its grounding assumptions discussed in the **Results** section may fail to include mechanisms that could manifest on timescales longer than the 80 minute imaging period. To address the question of model appropriateness on the timescales of our data, we compared our model to the following alternative model structures with AIC as the basis for quantitative comparison:

Case 1. Exclude 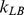: DTPA absorption in 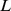, represented by rate constant 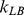, is not appreciable over the 80-minute functional imaging timescale and should not be regressed to achieve model fit

Case 2. Include 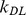: MCC in 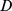, represented by rate constant 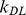, is significant over the 80-minute functional imaging study timescale

**Figure 9:**
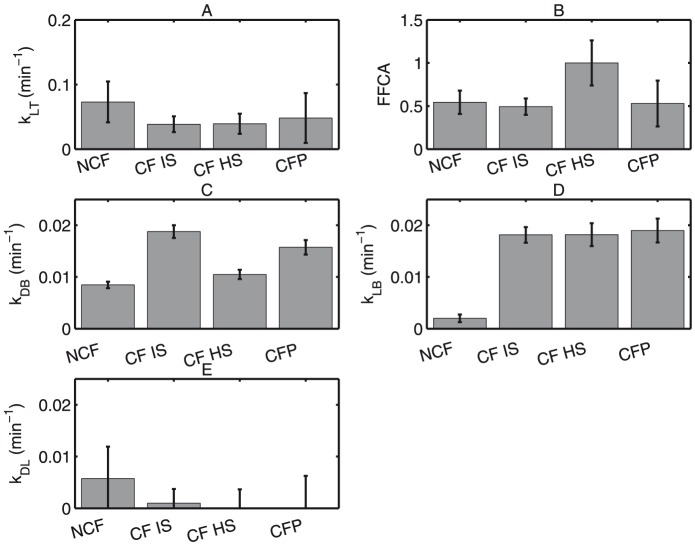
Least-squares solutions for model parameters in case 2 of Table 3 are shown for comparison to the selected model with 95% confidence intervals for four patient groups: Non-CF (NCF), CF HS, CF IS, and Pediatric CF (CP). (A) MCC rate constant (kLT) (min-1) reiterates that kLT is the same in all groups. (B) Fraction (FFCA) of large airway with functional MCC. (C) Peripheral lung absorptive rate constant (kDB) (min-1) and (D) large airway absorptive rate constant (kLB) are nearly identical to their analogs in the selected model. (E) MCC rate constant (kDL) (min-1) from D to L is clearly unidentifiable, and the nominal value is much smaller than kLT in each group. Confidence intervals are also widened in all parameters directly related to MCC.

The AIC values of each case above, in comparison to the model in Figure 2, are given in Table 4. These values demonstrate that the Figure 2 model displays the best combination of model fit and parsimony for the data set from [10]. Parsimony was especially important for model selection between the nominal case and Case 2, where the difference between AIC of the two cases was less than 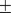
1%. However, it is important to again note that there may in fact be clearance mechanisms from 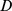, 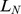, and 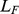 that are not accounted for in this model because of constraints on our ability to quantitatively resolve their effects with confidence over the timescale of imaging. This is illustrated graphically in Figure 9, where it is clear that we cannot confidently identify the parameter 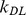 employed in Case 2 above.

**Table 4:** AIC Values for Various Model Structures.

	Model	Case 1	Case 2
NCF	−2546.28	−2535.65	−2543.38
CF HS	−2430.25	−2227.60	−2426.25
CF IS	−2775.62	−2287.07	−2775.57
CF Pediatric	−1858.37	−1597.47	−1854.37

The Akaike Information Criterion (AIC) is a metric for determining the most appropriate model for a system, where better fit is balanced against a penalty for overparameterization. Total AIC is shown as calculated from Equation 7 for all cases: 1) exclude 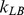 from model structure; 2) include 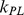 in model structure.

### Parametric Considerations

In non-CF subjects, FFCA is found to be 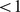. To our knowledge, the notion that not all of the surface of the conducting airways has functional cilia is a novel finding in humans. Hoegger *et al.* [23] recently report that even in the trachea, the most conductive airway, of newborn pigs without CF
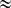
70% of particles clear in ten minutes and that those same particles are only actively moving 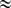
80% of the time. While this timeframe is considerably shorter than the one in our study, it has been established that particles can be retained in the conducting airways of non-CF animals for a potentially significant amount of time. It would be reasonable to assume that particles deposited deeper in the airways, like those in our study, would be retained longer. Thus we have further reason to believe, in addition to the *in vitro* findings presented here, that our estimation of FFCA as a model parameter is grounded in realistic physiology.

Our model asserts that the rate of DTPA absorption (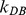) is increased in the peripheral lung (small airway and alveoli) compartment of patients with CF as compared to non-CF subjects. Hypertonic saline slows this rate to nearly non-CF levels. Previous *in vitro* studies have related DTPA hyperabsorption to liquid hyperabsorption [9]. It is assumed that DTPA hyperabsorption occurs via a paracellular mechanism, driven by increased paracellular liquid absorption, as DTPA has no known transcellular route by which to absorb. Therefore, it is likely that our current result reflects the basic liquid absorption defect associated with CF. The aerosol delivery techniques utilized to deliver the radiopharmaceuticals targeted large and small airways through a specialized breathing pattern. Additionally, transient correction of the absorption defect would be expected after administration of an inhaled osmotic therapy based on most current hypotheses of airway disease in CF [24]. However, we cannot completely exclude the possibility of alveolar effects that may be related to yet undocumented physiological differences between the alveolar regions of CF and non-CF lungs.

The rate at which DTPA absorbs in the large airways (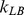) is similar to 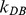 in the CF IS, CF pediatric and non-CF groups. However, while hypertonic saline decreases the value of 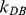 in patients with CF, it has no significant effect on 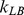. MCC is the dominant clearance mode in the large airways in both IS and HS cases. We have shown *in vitro* that HS modulates the FFCA of the airway epithelium and have shown *in vivo* that increased MCC can be attained by modulating FFCA without requiring change in MCC rate. The two rate constants in the central region (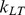 and 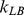) describe competing and correlated processes, whereas FFCA describes a more macroscopic phenomenon. Therefore, we would expect that a change in one rate constant in L would induce a corresponding change in the other. Between IS and HS days there is no change in 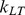 (the dominant process), so we would not expect a change in 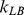 either. However, due to the increase in FFCA, total MCC increases in the central lung after HS inhalation, which results in less DTPA being present and able to absorb in accordance with its unchanged rate constant. In effect, the underlying rate of DTPA absorption is unchanged, but an increase in total MCC as a result of increased FFCA leads to less DTPA being available for absorption and a smaller total quantity of DTPA absorbed in the CF HS case. In the case of D, however, we have assumed there is no competing, certainly no governing, MCC term in D. Thus, conditions affecting D must result changes in 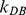.

### Study Limitations

A primary limitation of the functional imaging method utilized is its inability to uniquely discern airways and alveoli. Given the three dimensional nature of the lung architecture, small airways and alveoli are likely to be included in any zone analyzed via 2D imaging. The small sizes of these structures make them impossible to differentiate from each other through direct imaging, though other physiological measurements (such as 24 hr MCC) may help to differentiate their functional effects. The limited time scale of imaging (80 minutes) in the current study may have also limited assessment of small airway mucociliary clearance. Extended-duration imaging studies may yield additional data that can inform processes with slower rates and model parameters such as 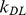, which was excluded from the Figure 2 model on AIC grounds.

### Physiological Implications

Our original study (see [10]) described significant differences in baseline whole lung DTPA absorption when comparing CF and healthy subjects. MCC was decreased in the CF groups, but not at statistically significant levels. In this study we find that MCC is decreased (in a statistically significant way) in the CF IS group for 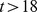 minutes. The difference between these two findings arises from differences in the analysis tools used in this work versus the endpoint analysis performed in [10]. The confidence intervals presented here are around the parameters of the model, as opposed to being derived from statistical analysis of a final retention value, as in [10]. Our parameters govern the entire time-course of clearance, and thus are informed by the entire dynamic of the data given in [10]. The resulting confidence-interval-derived envelopes in Figures 5 and 6 capture the uncertainty of each physiologically descriptive parameter in the model. This is achieved by simulating the model at all possible combinations of upper and lower CIs for each parameter and determining the extreme values of model prediction at every time point. Hence, the CIs about our parameters are used to generate physiologically, as opposed to empirically, informed predictions of clearance behavior in each subject group population. The narrowness of our parameter CIs indicates confidence in the model parameter estimates (in that they are well informed from the dynamic data in [10]) and generates narrow prediction envelopes in each case, indicating a physiological difference in MCC dynamics between patients with CF and non-CF subjects that is not found via our previous endpoint-analysis methods.

Response to HS inhalation was apparent in terms of both decreased absorption and increased MCC as compared to IS inhalation. These trends are reflected in our modeled result. Our model suggests that the observed increase in MCC following HS inhalation is due to an increase in FFCA rather than an increase in MCC rate, and a detailed analysis of this hypothesis using the *in vivo* imaging data is ongoing. The model also predicts that FFCA is reduced in patients with CF as compared to the healthy population. We used this prediction as a hypothesis about inducing a functional change *in vivo* that can be tested *in vitro*. To further elucidate this phenomenon, and validate that such behavior is possible, we performed *in vitro* studies with HBE cell cultures. A quantitative visual assay of ciliary movement demonstrated that a larger fraction of the cell culture surface was activated after the addition of hypertonic saline vs. isotonic saline.

### Clinical Relevance

Some previous imaging studies have shown significant differences in whole lung MCC between CF and non-CF subjects [25], [26] while others have not [4]. By ascribing a fixed mathematical structure to our model, we constrained the clearance curves to a single family of dynamical forms, which allows for more sensitive comparisons between the dynamical, and also endpoint, behavior present from one patient group to another. This ultimately increases our confidence in the model assertion that MCC is decreased in patients with CF and can be increased by hypertonic saline inhalation and, subsequently, that our model offers a better tool than statistical models for use in clinically gauging MCC.

Applications of this model may include the design of dosing regimens for agents targeting changes in MCC and/or absorption. For example, the timeline of the effects associated with an osmotic therapy (correction of liquid absorption defects) could be assessed independently of the resulting secondary effects (recruitment of functional ciliated airway, large and small airway clearance). Dosing timed in accordance with the duration of the counter-absorptive effect may provide more continuous clearance from the lungs and patient benefit, though outcome assessment is beyond the scope of the model at present. The model could also be used to resolve the pharmacodynamics of medications with subtle, complex, or multiple therapeutic effects, to the degree that these agents impact MCC and absorption.

### Conclusions

Our physiologically motivated compartmental model is able to reproduce the clearance behavior of both large particle (Tc-SC) and small molecule (DTPA) radiolabeled probes as informed by functional imaging data. The model asserts that liquid absorption rate is increased in the peripheral lung of patients with CF as compared to non-CF subjects, and that hypertonic saline will decrease the rate of absorption in patients with CF to near non-CF levels. Our model also attributes increased MCC induced by hypertonic saline treatment to an increase in airway surface area with functional ciliary clearance, as opposed to an increased rate of clearance. The physiology inherent to the model structure and parameterization allow for increased sensitivity in gauging the effects of hypertonic saline *in vivo* in a real-time manner. This model should, therefore, be extendable to other treatments, both present and future, that target the liquid hyperabsorption and MCC deficiency present in cystic fibrosis.

## Materials and Methods

### Functional Imaging Methods

Data from previously reported clinical imaging studies were utilized [10]. These studies included both adult (n = 12) and pediatric (n = 9) patients with CF and healthy controls (n = 9). Raw imagining data from this study can be found in Table S1. An aerosol based functional imaging technique that measures the clearance of two different radiopharmaceutical probes from the lungs over 80 minutes was used. Indium-111-labeled diethylene triamine pentaacetic acid (DTPA) is a small molecule probe (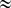
500 Da) that is cleared from the lung through both absorption and MCC while Technetium-99m-labeled sulfur colloid (Tc-SC) is a particle probe (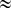
300nm) that is cleared only by MCC. DTPA absorption is calculated by subtracting the Tc-SC clearance rate (MCC) from the total In-DTPA clearance rate. Both probes were mixed and delivered together via nebulizer in the same liquid aerosol. All patient studies were approved by the University of Pittsburgh Institutional Review Board (clinicaltrial.gov numbers NCT01223183 and NCT01486199).

The aerosolized probes were delivered simultaneously by nebulizer using a technique that deposited aerosol primarily in the airways [10]. Their clearance (normalized decrease in radioactive counts over time) was then independently assessed over 80 minutes using dynamic planar scintigraphy. Whole, peripheral and central lung zones were considered, where the central zone was defined as a rectangle with 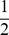 the height and width of the whole lung region of interest postioned along the medial lung border and centered vertically. At t = 10 minutes (11th imaging frame) subjects inhaled nebulized saline treatments for 10 minutes. Adult patients with CF inhaled 7% hypertonic saline (HS, n = 11) on one testing day and isotonic saline (IS) on the other. Control and pediatric CF subjects performed a single testing day with isotonic saline. Previous *in vitro* studies have demonstrated a relationship between DTPA and ASL absorption. Since DTPA has no known route for intracellular transport this relationship is presumably due to the contribution of paracellular liquid flows. Changes in DTPA absorption in response to osmotic therapies have previously been demonstrated both *in vitro* [9] and *in vivo* [10]. Analysis of the Tc-SC and DTPA retention curves as well as DTPA absorption plotted with a logarithmic ordinate showed that the data did not follow a monoexponential profile, which guided selection of the model structure and kinetics necessary to accurately reproduce the data.

### Initial Model Structure

Sakagami [15] proposed a robust modeling scaffold for inhaled pharmaceuticals that takes into account the regional pharmacokinetic differences between the large conducting airways (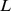) and the distal lung (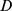), consisting of smaller airways and alveoli. Analysis of the corresponding system of compartment-based ordinary differential equations (ODEs) yielded a bi-exponential retention curve for Tc-SC as proposed by Byron [14]. However, this model structure could not be reconciled with the whole of the data used herein (see **Discussion**).

### Model Parameter Estimation

Regression of model parameters, regardless of model structure, was performed sequentially as follows. Model parameters were estimated using nonlinear least-squares regression (via 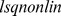 in MATLAB, ©2013, The MathWorks, Natick, MA) between model predictions and experimental data. The experimental design allowed for sequential estimation of the MCC and absorption rate parameters as follows:

1. The parameters that govern MCC (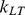 and FFCA) were fit to the Tc-SC retention data.
2. The regressed MCC parameters were then fixed, and the absorption rate parameters (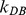 and 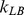) were fit to the absorption curve.
3. Parameter uncertainty was calculated using the 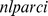 function in MATLAB (©2013, The MathWorks, Natick, MA) to obtain confidence intervals on all model parameters.

### Model Analysis

The assumptions above were tested individually, and in combination, for appropriateness using the Akaike Information Criterion (AIC). Akaike [21] originally proposed that model appropriateness could be assessed according to the equation: 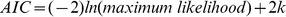
(6)


Where a Gaussian distribution represents the maximum likelihood of the data and k is the number of free parameters in the model. Thus, the model with the least value of AIC is deemed most appropriate in terms of model fit and parsimony. Yamaoka and colleagues [27] further elaborated on this information criterion by proposing that for a process with Gaussian error the AIC can found from the equation: 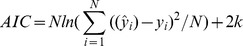
(7)


Where 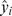 is the model fit and 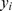 is the data value at the 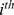 image of our N = 80n set because there are 80 images per patient and n patients per study group.

### *In Vitro* Assessment of Ciliary Activation

We developed an *in vitro* imaging assay to measure the fraction of the epithelium that has functional MCC in order to test our hypotheses that FFCA is decreased in CF and that hydration can recruit areas of otherwise non-functional cilia. Fully differentiated human bronchial epithelial (HBE) cell cultures were viewed using a phase-contrast objective (Nikon Eclipse TI). These cultures were derived from lungs removed at the time of lung transplantation and prepared using previously described methods approved by the University of Pittsburgh IRB [28]. A series of 10 images was taken of each culture that allowed for the determination of the change in pixel intensity between successive images in the stack. Ciliary beat can be observed under these conditions and is characterized by a change in light intensity measured by the camera. This change in light intensity can be translated to a change in pixel intensity in the images obtained when placed in sequence to form a movie of ciliary motion (see Figure S2 and Video S1). The average change in pixel intensity of all cultures was determined in both CF lines (n = 6) and a non-CF line. Average changes in the intensity of each pixel from 12 untreated cultures from the non-CF line were measured and the average change in intensity over all pixels was used as a baseline measurement of functional ciliary movement. Pixels in each stack with average change in intensity greater than the non-CF baseline were said to have functional ciliary movement. To test the effect of osmotic agents, 5 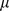
L of either isotonic (300 mOsm) or hypertonic (600 mOsm) saline was added to the apical surface of cells prior to imaging (see Table S2 for data). To test for saturating effects of hydration, we sequentially added Dulbecco's Modified Eagle Medium (DMEM) from the basolateral bath to the apical surface of the cultures as follows: 10 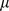
L of DMEM was added to the apical surface of the cells from the basolateral bath before the cells were imaged, and an additional 10 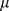
L of basolateral DMEM was added to the apical surface prior to a second, identical, imaging routine (see Table S3 for data).

## Supporting Information

Figure S1
**Shown is the initial posterior frame of a Tc-SC imaging series.** The dotted outline of the right lung is a tracing of the outline of the lung as it appears in a transmission scan. The filled in blue rectangle is the 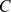 ROI. The remaining lung ROI represents 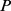.(TIF)

Figure S2 **A pseudo-color image of non-CF HBE cells under 10× enhancement is shown.** The viewing frame is identical to that in Video S1. Darker (more blue) regions show areas with lower average change in pixel intensity during Video S1 whereas lighter (more yellow) regions show areas with higher average change. In combination with Video S1 we show that regions that display little visible movement are more blue, and the regions with visible motion, particularly the hurricane regions, are more yellow.
(TIF)

Table S1 **Raw Tc-SC and In-DTPA counts in whole lung ROI and central lung ROI over the length of imaging.**
(PDF)

Table S2 **FFCA in CF HBE cells is shown at baseline compared to a 5 ul addition of either isotonic or hypotonic saline.**
(PDF)

Table S3 **FFCA in CF HBE cells is shown at baseline, after one 10 ul addition of basolateral DMEM, and after a second, sequential addition of basolateral DMEM.**
(PDF)

Video S1 **A video of ciliary motion in non-CF HBE cells under 10× phase-contrast enhancement is shown.** The viewing frame is identical to that in Figure S2. Three hurricane type regions of ciliary motion are visible in the field of view. In combination with Figure S2 we show that regions that display little visible movement have lower average change in pixel intensity, and the regions with visible motion, particularly the hurricane regions, have higher average change in pixel intensity.
(AVI)
