# Supplementary figures and images for: A Physiologically-Motivated Compartment-Based Model of the Effect of Inhaled Hypertonic Saline on Mucociliary Clearance and Liquid Transport in Cystic Fibrosis
Source: PLoS One. 2014 Nov 10;9(11):e111972. doi: 10.1371/journal.pone.0111972 (PMC4226497; doi:10.1371/journal.pone.0111972)

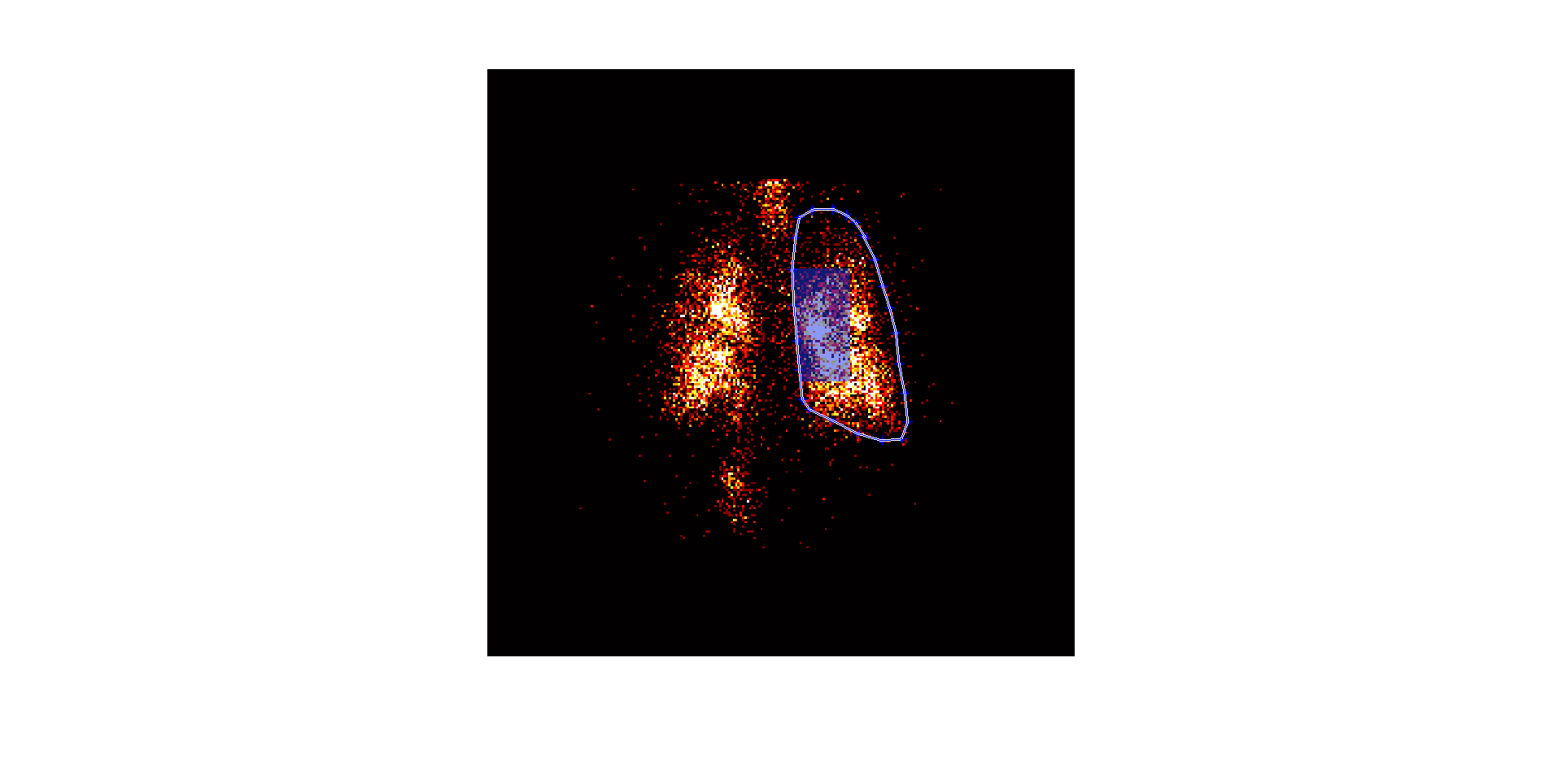

Supplement: Figure S1 — Shown is the initial posterior frame of a Tc-SC imaging series. The dotted outline of the right lung is a tracing of the outline of the lung as it appears in a transmission scan. The filled in blue rectangle is the ROI. The remaining lung ROI represents . (TIF) [file pone.0111972.s001.tif]
