# Supplementary material for: A Physiologically-Motivated Compartment-Based Model of the Effect of Inhaled Hypertonic Saline on Mucociliary Clearance and Liquid Transport in Cystic Fibrosis
Source: PLoS One. 2014 Nov 10;9(11):e111972. doi: 10.1371/journal.pone.0111972 (PMC4226497; doi:10.1371/journal.pone.0111972)
Supplement: Table S1 — Raw Tc-SC and In-DTPA counts in whole lung ROI and central lung ROI over the length of imaging. (PDF) [file pone.0111972.s003.pdf]

Whole Lung Tc-SC Counts (CF IS)

	Patient											
Frame	1	2	3	4	5	6	7	8	9	10	11	12
1	333.9	823.8	880.0	3543	1717.7	2177.9	2468.4	2592.5	4489.3	2256.2	6528.4	2740.6
2	305.2	700.4	755.7	3356	1673.8	2017.0	2665.4	2706.2	4531.7	2211.9	6551.2	2552.6
3	324.1	868.8	835.7	3451	1621.0	2076.8	2544.8	2660.9	4707.7	2338.8	6437.3	2577.5
4	381.4	907.2	839.0	3486	1719.5	2072.9	2565.4	2517.8	4544.9	2272.5	6305.7	2500.3
5	338.1	751.0	791.4	3313	1654.7	2121.8	2589.4	2431.5	4676.5	2240.1	6396.1	2572.4
6	347.5	728.5	838.3	3162	1683.6	2169.6	2509.4	2444.8	4583.3	2166.2	6129.3	2557.4
7	338.8	737.7	855.2	3293	1755.4	1967.0	2440.1	2392.8	4287.2	2092.8	6198.5	2481.9
8	318.9	833.7	826.0	3330	1677.6	1941.0	2492.0	2409.1	4357.8	2216.1	6084.0	2394.6
9	361.1	683.3	876.0	3055	1667.7	2001.6	2364.8	2388.6	4473.4	2066.4	5953.5	2323.1
10	325.1	710.1	902.0	3167	1642.7	1978.0	2246.7	2370.2	4279.4	2164.8	5987.5	2342.8
11	341.0	695.6	784.3	3019	1644.7	1902.9	2158.9	2346.2	4193.9	2054.0	5651.3	2479.8
12	342.2	628.1	879.4	3266	1745.1	1811.6	2126.5	2304.2	4178.9	2186.6	5531.1	2309.8
13	366.6	657.1	785.7	3021	1635.5	1981.3	2244.7	2293.6	4114.1	2043.4	5281.1	2149.9
14	351.6	637.4	761.4	3218	1690.5	1877.8	2256.8	2254.0	4214.5	2200.2	5237.8	2306.6
15	313.4	658.4	778.5	3113	1673.1	1946.2	2222.8	2298.0	4248.1	2222.4	5268.4	2124.6
16	311.4	649.2	817.4	3004	1674.0	1786.0	2134.2	2404.3	4178.6	2150.4	5121.0	2173.6
17	317.2	550.3	819.7	2973	1767.4	1978.7	2161.6	2306.5	4172.7	2007.1	5080.0	2064.0
18	296.7	617.7	799.2	3002	1686.5	1823.2	2084.0	2263.9	4118.2	2082.1	5102.6	2205.2
19	305.7	622.5	734.9	3116	1595.4	1863.7	2180.9	2341.9	4172.7	2150.4	5105.4	2106.1
20	316.7	579.7	779.2	3102	1610.4	1847.8	2215.7	2339.3	4282.4	2085.4	5204.9	2186.4
21	310.8	618.7	754.3	2988	1618.5	1826.4	2150.1	2333.6	4216.9	2156.3	5072.8	2130.8
22	318.4	592.3	763.6	2926	1572.8	1704.9	2150.6	2232.8	4190.0	2163.2	5109.8	2127.3
23	313.6	638.0	760.0	3020	1585.0	1818.8	2194.6	2250.0	4202.3	2168.4	5341.8	2170.0
24	282.6	562.6	793.6	2929	1574.5	1832.5	2208.4	2353.0	4306.9	2080.7	4875.2	1987.8
25	381.5	564.0	766.4	3002	1640.7	1834.7	2121.5	2199.9	4107.8	2135.6	4883.8	2111.8
26	250.0	636.4	765.9	2817	1543.8	1834.4	2138.7	2171.0	4219.1	2065.0	4866.3	2200.7
27	327.1	601.6	777.9	3012	1633.4	1772.6	2024.8	2341.3	4342.9	2199.0	4955.6	2080.8
28	328.1	573.8	840.2	2955	1634.1	1781.5	2038.8	2271.1	4185.0	2119.2	4977.4	2097.1
29	303.9	533.4	819.2	2942	1660.1	1786.9	2216.3	2152.3	4180.6	2115.7	4899.9	2133.2
30	275.6	546.3	715.5	2941	1603.3	1820.1	1979.1	2190.2	4216.4	2104.3	5014.1	2038.3
31	350.7	604.1	784.3	2911	1636.0	1786.3	2131.1	2334.2	4188.9	2081.0	4883.1	2105.6
32	347.3	577.7	734.4	2956	1483.0	1677.5	2043.2	2354.5	4159.8	2245.4	4826.3	2073.1
33	287.7	578.1	812.9	2798	1675.1	1828.6	1980.9	2240.3	4319.3	2036.0	5014.4	2146.0
34	366.9	534.7	795.5	2816	1583.8	1809.7	2158.7	2314.3	4340.7	2097.9	4997.0	2034.5
35	361.2	565.7	815.9	2797	1625.3	1740.0	1990.1	2310.7	4107.1	2063.7	5088.6	2069.8
36	329.2	587.0	749.9	2844	1462.9	1823.1	2056.2	2443.0	4490.3	2027.1	4842.6	1914.0
37	334.7	541.9	697.5	2938	1561.2	1759.7	2006.2	2229.2	4176.2	1984.5	4819.1	2139.8
38	338.0	520.6	799.0	2725	1600.7	1818.5	2058.1	2473.6	4275.3	2022.7	4796.1	1974.7
39	313.2	533.5	785.2	2854	1604.7	1782.9	2099.4	2385.7	4225.5	1923.7	4873.3	2053.5
40	342.8	575.6	779.6	2776	1647.6	1732.3	2089.5	2382.7	4329.5	1952.1	4962.3	2076.3

41	291.7	518.5	725.5	2800	1644.7	1777.5	1930.6	2377.4	4223.8	2037.0	4782.2	2064.2
42	282.5	577.9	698.1	2770	1656.1	1820.6	2025.4	2313.4	4107.7	2017.8	4806.4	1990.3
43	326.6	595.6	724.5	2695	1639.5	1759.7	2073.3	2354.0	4294.8	1923.4	5067.1	1991.7
44	361.2	538.8	717.1	2701	1564.6	1732.7	1987.9	2301.3	4205.2	1895.0	4936.6	2056.9
45	301.4	567.3	741.0	2692	1604.3	1773.1	2072.6	2225.3	4220.3	1972.5	4816.2	1904.5
46	236.3	485.6	765.4	2949	1616.9	1761.6	2142.7	2364.7	4263.2	2015.1	4913.2	2005.9
47	278.6	546.0	726.2	2699	1582.6	1833.8	1921.4	2226.4	4332.3	2052.6	4712.0	1978.1
48	295.9	516.6	698.2	2777	1554.1	1746.9	1985.6	2443.9	4224.8	1907.6	4922.7	1830.1
49	292.5	559.8	761.3	2656	1557.6	1797.4	2116.5	2380.4	4258.0	1995.7	4894.7	1879.8
50	298.0	543.9	725.4	2696	1604.2	1690.0	2100.7	2460.8	4374.9	1908.9	4926.6	1870.7
51	299.6	560.7	739.1	2655	1542.8	1634.7	2029.7	2346.7	4205.1	1999.2	4823.8	2022.2
52	249.9	483.8	718.7	2511	1604.6	1840.5	2000.5	2265.6	4409.1	1979.3	4934.5	1977.5
53	315.8	481.0	685.5	2573	1538.1	1755.0	2010.2	2238.6	4230.6	1785.5	4783.7	1984.6
54	299.8	539.8	618.8	2472	1601.6	1786.8	2004.0	2353.3	4140.5	1977.7	4660.8	1864.9
55	309.8	476.4	694.1	2516	1570.7	1812.5	2025.7	2457.6	4111.4	1751.0	4703.1	1950.2
56	250.6	497.1	672.0	2473	1609.4	1667.4	1916.3	2287.1	4165.3	1954.9	4660.5	1909.8
57	329.9	561.7	696.7	2468	1556.6	1797.7	1975.8	2410.7	4019.8	1873.2	4620.6	1989.4
58	345.7	428.2	702.2	2490	1497.4	1852.8	2034.6	2375.0	4263.4	1758.7	4541.7	2073.4
59	304.3	424.6	698.5	2529	1630.0	1616.3	2024.6	2275.9	4119.5	1808.7	4404.0	1912.8
60	206.0	442.6	733.5	2552	1610.2	1650.7	2136.5	2295.1	4230.0	1927.7	4605.0	1983.0
61	280.6	436.9	695.8	2633	1573.0	1691.4	1999.9	2392.6	4341.8	1865.6	4431.2	2012.7
62	330.9	471.7	728.5	2497	1465.1	1790.7	2032.6	2321.7	4589.8	1823.1	4671.6	1842.6
63	256.1	493.8	735.0	2555	1501.1	1643.7	2011.5	2337.9	4396.7	1902.1	4570.5	1986.9
64	271.0	467.7	665.1	2413	1485.6	1722.9	1911.1	2489.5	4247.7	1924.4	4552.2	1878.5
65	248.3	429.8	658.2	2327	1565.1	1738.6	1915.3	2201.4	4431.1	1788.2	4649.1	1989.4
66	271.7	492.1	689.7	2364	1616.4	1589.8	2013.3	2385.7	4289.3	1942.5	4470.6	1950.7
67	241.5	393.6	598.6	2411	1563.6	1663.5	2104.6	2380.9	4403.0	1695.1	4654.0	1819.0
68	276.0	496.1	693.7	2474	1564.6	1724.1	2003.7	2222.0	4270.6	1688.5	4570.8	1932.5
69	323.7	497.0	639.8	2334	1637.5	1803.7	1938.3	2427.4	4327.4	1822.8	4668.1	1922.1
70	271.5	489.1	715.0	2464	1484.1	1585.5	1993.6	2352.2	4372.0	1786.5	4686.3	1835.6
71	315.5	504.0	757.9	2233	1521.8	1681.7	1997.6	2277.0	4187.6	1859.8	4635.9	1951.0
72	275.9	462.3	674.9	2399	1621.5	1671.9	1992.0	2297.0	4318.1	1789.1	4611.2	1735.7
73	249.9	541.3	675.2	2358	1524.9	1700.7	2145.5	2280.6	4426.4	1813.0	4677.6	1941.2
74	278.8	516.6	723.8	2276	1567.5	1693.3	2020.3	2336.4	4182.1	1674.5	4405.6	2015.7
75	329.0	523.1	728.2	2289	1580.0	1591.5	1977.8	2318.6	4267.1	1716.3	4673.3	1945.9
76	293.9	433.9	627.4	2174	1573.8	1665.2	2008.4	2326.4	4193.9	1738.7	4454.8	1872.5
77	276.1	482.1	698.0	2194	1475.2	1646.1	1939.8	2398.1	4174.7	1639.3	4642.7	1880.4
78	270.5	498.7	643.9	2289	1634.8	1621.0	1981.2	2325.8	4148.5	1747.3	4638.0	1837.9
79	210.6	471.7	664.9	2250	1578.3	1569.7	1973.5	2198.8	4147.8	1738.6	4678.4	1829.6
80	241.9	486.6	680.4	2260	1622.6	1644.7	2004.7	2353.3	4147.1	1641.3	4676.5	1979.7

Central Lung Tc-SC Counts (CF IS)

	Patient											
Frame	1.0	2.0	3.0	4	5.0	6.0	7.0	8.0	9.0	10.0	11.0	12.0
1	156.2	354.9	325.4	2065	657.1	833.5	1316.2	1136.6	1716.9	1211.3	2839.8	1517.1
2	141.9	276.9	293.5	1932	685.1	749.0	1425.5	1143.7	1758.6	1043.8	2864.4	1239.7
3	182.1	347.1	318.8	1978	640.4	798.0	1321.4	1149.3	1777.9	1208.8	2723.4	1307.7
4	185.4	387.7	302.7	2025	701.2	794.5	1325.1	1035.7	1570.0	1182.1	2588.2	1215.6
5	166.1	321.5	307.5	1910	638.3	797.0	1215.0	1076.9	1762.6	1169.9	2695.6	1281.8
6	166.5	301.4	324.7	1890	659.5	828.9	1131.7	1101.6	1704.3	1061.0	2436.3	1285.8
7	162.3	288.5	354.0	1838	650.9	689.7	1100.4	988.3	1473.9	1140.5	2394.8	1191.7
8	160.9	337.7	320.7	1899	658.7	652.1	1179.4	933.7	1524.5	1095.8	2315.0	1131.9
9	176.6	277.2	322.3	1758	634.3	664.5	1086.1	1028.1	1561.7	1100.2	2164.1	1139.9
10	153.3	292.1	321.3	1780	618.9	654.7	1051.7	1057.1	1461.9	1120.9	2016.2	1046.8
11	147.2	262.3	278.4	1648	632.6	552.4	938.5	1062.9	1386.2	1061.4	2101.8	1058.0
12	147.5	232.4	315.0	1844	663.1	542.4	952.4	973.1	1459.1	1137.0	1942.7	1068.6
13	156.4	235.2	268.3	1722	627.6	687.4	1015.9	980.9	1342.8	1041.3	1820.7	979.4
14	173.4	225.1	280.5	1778	639.5	637.9	1037.5	883.6	1419.6	1138.0	1801.1	1111.4
15	136.5	228.3	304.7	1752	590.9	635.8	977.7	955.1	1583.4	1102.9	1795.6	952.7
16	142.7	222.1	298.5	1760	628.1	630.8	975.5	1018.4	1558.3	1122.6	1683.1	962.2
17	136.3	192.9	330.8	1681	645.3	691.3	989.2	970.3	1528.4	1052.7	1681.9	951.9
18	171.9	238.4	278.5	1600	677.0	641.8	963.7	1023.1	1512.3	1052.2	1608.1	1007.5
19	170.5	194.4	277.5	1742	686.3	639.6	1061.0	1065.4	1520.0	1139.7	1454.4	928.0
20	165.0	217.1	278.0	1672	649.2	641.7	1010.5	1039.5	1562.8	1083.6	1626.6	952.3
21	203.9	244.9	285.3	1709	606.2	621.0	1018.9	1006.8	1518.1	1047.3	1718.8	938.6
22	136.3	251.0	274.9	1585	672.4	551.8	1005.1	1033.9	1580.4	1135.3	1815.8	965.2
23	149.6	266.6	277.5	1768	655.5	576.1	988.7	971.7	1605.1	1142.2	1865.0	964.9
24	141.0	199.4	315.9	1728	639.7	591.2	1058.5	960.7	1596.3	985.2	1500.9	818.6
25	187.1	235.1	272.3	1739	655.6	616.8	909.6	990.2	1553.1	1033.4	1521.6	917.9
26	143.9	211.9	278.5	1647	659.2	635.0	1025.2	915.6	1510.6	974.6	1640.8	924.7
27	187.1	224.2	277.6	1804	690.5	602.4	906.9	930.2	1666.1	1045.4	1650.1	892.8
28	178.5	225.6	284.6	1711	725.0	556.0	920.4	860.2	1581.6	1065.6	1677.0	964.3
29	183.6	214.2	291.2	1763	725.3	578.0	996.5	822.4	1533.0	1031.4	1534.8	928.9
30	142.5	173.4	259.8	1736	645.2	559.7	878.8	883.4	1521.4	976.4	1619.3	962.1
31	187.2	236.2	293.1	1723	637.0	596.6	936.0	1035.5	1475.7	1050.6	1491.9	904.6
32	175.5	222.9	246.2	1800	557.9	546.6	917.9	896.4	1495.4	1071.7	1577.8	939.5
33	171.3	202.5	306.5	1688	600.1	591.2	812.8	867.2	1568.8	950.3	1573.8	943.2
34	171.2	188.3	299.3	1633	580.6	619.2	945.1	929.6	1530.5	983.3	1580.9	843.8
35	169.0	204.1	281.8	1652	590.0	534.7	892.9	983.8	1443.4	1057.7	1614.9	848.9
36	147.2	212.8	254.2	1680	534.6	621.5	958.6	1041.4	1606.1	967.3	1489.9	819.6
37	140.1	172.0	245.6	1689	588.9	595.4	930.7	914.8	1466.5	949.0	1535.4	938.5
38	161.9	206.3	295.5	1623	628.1	575.8	931.2	1002.2	1572.2	923.9	1642.0	847.7
39	180.5	216.5	261.9	1691	569.8	567.1	955.1	948.7	1462.5	896.7	1668.7	859.8
40	181.5	210.4	274.8	1631	596.6	573.0	966.0	1035.6	1512.3	905.6	1631.9	939.7

41	162.1	192.4	245.8	1652	553.1	563.1	847.6	966.3	1396.6	938.9	1533.0	944.8
42	172.6	186.2	240.3	1693	498.5	645.2	857.0	983.1	1447.3	923.4	1559.0	990.5
43	178.1	203.2	243.8	1650	548.4	598.2	955.2	997.1	1481.2	929.9	1710.4	885.3
44	176.4	188.1	238.3	1681	560.7	596.5	895.2	954.4	1530.4	886.4	1638.2	945.6
45	171.3	200.9	264.8	1648	662.6	629.6	942.8	941.9	1455.9	914.6	1656.9	847.5
46	144.9	196.4	243.0	1756	704.5	576.3	934.6	978.0	1424.2	895.3	1704.1	911.5
47	182.6	215.7	274.9	1627	697.9	552.9	865.0	885.2	1457.6	969.9	1642.2	900.1
48	156.8	183.8	262.8	1744	683.6	577.9	877.4	893.5	1386.7	799.9	1688.5	880.3
49	173.2	197.5	275.7	1607	639.6	585.1	898.8	831.3	1461.7	896.3	1694.3	880.3
50	181.8	195.2	243.8	1695	708.7	567.5	955.2	857.7	1519.2	863.2	1695.6	897.1
51	165.6	211.8	234.5	1631	609.9	533.3	937.6	836.3	1440.3	925.3	1587.6	916.1
52	160.8	173.3	278.3	1591	720.1	574.1	867.0	848.4	1444.5	896.2	1604.6	916.7
53	177.6	225.3	249.0	1608	711.4	598.1	880.0	797.4	1467.2	836.8	1644.8	931.5
54	195.7	227.9	176.4	1539	674.6	635.1	896.6	909.6	1271.0	882.6	1480.0	849.1
55	163.4	188.4	214.8	1562	702.4	597.9	837.2	924.7	1344.2	847.5	1322.5	894.5
56	161.4	167.7	183.5	1531	694.0	565.1	884.5	894.0	1337.2	888.8	1485.1	951.2
57	191.9	239.6	276.3	1464	633.2	543.9	791.9	982.6	1395.4	911.5	1401.8	945.9
58	186.5	220.5	275.7	1553	650.1	618.4	910.4	857.3	1442.0	808.2	1340.9	898.3
59	160.6	204.5	206.0	1479	679.3	540.9	930.4	884.6	1628.4	848.1	1373.4	843.4
60	110.8	232.6	240.4	1552	582.0	476.8	912.6	845.6	1690.9	889.8	1429.8	836.5
61	111.3	201.3	254.5	1580	637.5	539.2	879.2	957.8	1477.8	859.2	1335.1	930.7
62	172.7	202.5	237.6	1432	580.5	548.9	891.9	884.0	1641.8	777.7	1482.9	874.2
63	183.7	180.4	248.7	1502	647.0	490.7	833.2	865.4	1512.2	849.7	1395.6	910.3
64	152.1	233.2	232.5	1417	643.1	530.3	860.3	888.4	1613.6	866.1	1387.1	903.1
65	141.6	191.5	252.2	1467	647.8	582.0	776.8	755.7	1638.0	807.0	1411.1	840.6
66	141.0	227.0	219.6	1370	643.9	466.9	882.0	898.4	1582.5	875.3	1382.4	849.6
67	132.6	197.2	224.0	1363	673.2	544.4	927.4	861.6	1559.0	668.4	1422.8	813.9
68	173.6	196.1	220.9	1414	574.6	490.9	857.7	835.8	1519.0	786.8	1390.5	852.8
69	186.4	212.7	227.6	1347	679.8	605.4	865.5	922.2	1543.9	854.4	1399.1	896.4
70	143.2	211.4	236.4	1397	573.2	443.9	887.0	875.6	1585.6	789.8	1396.3	848.3
71	179.9	222.6	258.0	1257	564.0	412.5	912.1	844.1	1503.1	835.7	1467.6	878.2
72	158.8	206.1	256.4	1368	595.7	455.5	889.2	888.8	1527.6	806.1	1420.4	844.1
73	117.1	240.1	204.7	1340	577.1	433.9	934.1	863.5	1452.5	786.6	1571.5	876.4
74	153.9	191.7	258.5	1278	553.0	410.9	847.4	937.5	1316.9	710.4	1425.0	945.8
75	174.8	214.5	219.9	1299	576.0	403.0	888.3	869.0	1382.4	742.0	1461.9	894.3
76	162.9	178.9	180.2	1226	595.0	381.8	921.7	866.5	1315.7	833.0	1380.7	868.4
77	135.6	177.6	236.0	1219	567.8	414.9	846.2	997.5	1373.8	700.0	1531.6	819.7
78	137.9	199.5	232.1	1293	564.6	414.7	895.7	930.4	1444.4	765.0	1460.5	804.6
79	97.2	141.8	237.2	1222	588.6	353.0	830.9	826.7	1452.5	767.8	1344.2	855.1
80	96.1	160.7	229.2	1252	604.6	410.8	873.5	941.8	1368.6	698.1	1488.2	913.8

Whole Lung In-DTPA Counts (CF IS)

	Patient											
Frame	1	2	3	4	5	6	7	8	9	10	11	12
1	81.8	130.8	154.0	860.1	259.2	299.7	406.7	367.1	814.3	393.9	1567.9	539.5
2	77.4	135.8	133.0	870.3	291.3	320.7	399.7	345.1	848.5	355.9	1545.1	512.6
3	82.2	98.9	112.1	872.4	261.3	289.7	371.8	408.2	789.2	367.0	1467.4	511.7
4	85.3	90.9	134.1	755.5	248.4	273.8	415.9	363.2	753.9	345.0	1450.6	474.7
5	74.3	113.9	119.1	776.7	274.4	258.8	390.9	329.3	771.5	344.1	1423.8	539.9
6	71.1	101.9	127.1	863.9	252.5	271.9	362.0	321.5	739.4	395.2	1368.0	439.9
7	69.7	112.9	129.2	817.0	276.5	226.9	310.0	310.0	688.4	366.2	1349.2	450.9
8	60.1	102.9	141.2	797.1	250.5	265.0	337.1	286.0	723.6	358.3	1370.5	445.0
9	53.1	100.0	129.2	731.1	290.6	223.9	295.1	291.9	710.7	377.4	1335.7	430.1
10	84.9	112.0	133.2	760.3	276.7	189.9	241.0	266.9	676.8	329.4	1276.8	470.2
11	81.0	94.0	126.2	711.3	241.7	230.0	274.1	275.9	614.8	341.4	1141.8	421.2
12	73.6	97.0	110.2	771.6	244.7	237.1	221.1	262.9	651.9	376.6	1137.9	451.3
13	65.5	87.0	139.3	752.7	237.7	187.0	249.2	262.0	650.1	324.5	1178.2	412.3
14	68.6	91.0	121.3	738.8	224.7	225.1	248.2	241.2	621.1	313.6	1045.1	428.4
15	55.7	95.0	120.3	718.9	280.9	219.2	236.2	268.3	614.2	296.6	1081.4	383.4
16	53.7	96.1	102.3	761.1	232.8	184.1	239.3	280.4	603.3	330.7	1065.5	361.0
17	55.8	75.0	122.4	742.2	221.8	210.2	240.3	275.2	613.4	347.8	1020.6	379.1
18	45.5	102.1	106.3	723.2	256.4	186.2	203.2	254.0	596.4	321.8	1019.8	357.1
19	84.1	84.1	133.4	739.0	233.4	193.2	220.3	246.4	613.6	349.9	1037.0	347.1
20	48.2	75.1	102.4	781.7	214.5	202.3	202.3	256.5	565.5	290.8	897.7	368.3
21	47.2	65.0	93.3	740.7	254.7	202.3	194.3	252.5	598.8	293.9	993.2	323.2
22	71.3	77.1	130.5	745.8	222.6	170.2	184.3	233.5	577.8	332.1	927.1	356.3
23	65.3	74.1	103.4	710.8	215.0	186.3	170.3	220.1	593.9	325.3	934.3	347.4
24	71.3	65.1	112.5	645.7	238.2	169.3	188.4	258.7	511.7	349.4	931.4	354.5
25	62.3	83.2	123.5	666.9	230.2	150.2	182.4	229.6	586.1	300.3	940.6	343.1
26	60.1	73.1	104.5	711.2	212.1	165.3	197.5	221.6	551.1	325.5	895.6	349.2
27	54.1	82.2	94.4	695.2	209.2	181.4	171.4	216.6	531.1	335.6	823.4	310.0
28	61.1	67.1	105.5	673.2	226.3	142.3	180.5	230.7	612.5	323.6	859.7	324.2
29	74.2	49.0	127.6	710.5	218.3	177.5	198.6	184.5	559.4	264.3	846.8	296.1
30	67.1	71.2	111.6	650.3	223.3	142.3	156.4	216.7	543.4	337.7	844.9	280.0
31	76.2	94.3	98.5	622.3	181.2	150.4	161.5	196.6	579.7	286.5	879.3	285.1
32	69.0	60.1	109.6	658.0	215.4	188.6	143.4	214.8	598.9	296.6	871.4	291.2
33	64.4	64.2	112.6	633.4	210.4	175.6	130.3	196.7	515.5	319.8	840.4	338.5
34	68.4	68.2	110.6	672.7	195.1	145.4	155.5	203.8	511.6	312.8	861.6	271.2
35	60.4	67.2	108.7	637.6	222.3	143.5	133.4	197.8	560.0	314.9	829.6	333.6
36	58.4	54.1	88.5	650.8	201.2	149.5	138.5	224.0	515.8	299.8	819.7	300.5
37	72.5	80.3	106.7	661.0	268.3	114.3	138.5	205.5	491.7	297.7	825.8	287.4
38	78.5	78.3	112.7	625.9	201.9	139.5	132.5	196.5	458.6	287.7	810.9	266.3
39	47.7	68.3	87.6	619.9	206.0	160.7	151.6	217.7	477.8	303.8	847.3	255.3
40	63.8	68.3	88.6	613.0	210.0	140.6	109.3	204.6	478.9	292.8	765.8	267.4

41	62.0	60.2	133.9	646.3	207.1	123.5	112.4	189.5	470.9	254.6	772.0	263.5
42	34.9	72.3	108.8	663.6	224.2	110.4	118.5	211.7	431.7	286.9	787.3	246.4
43	66.3	59.2	77.6	621.4	150.7	162.8	127.5	222.8	445.9	264.7	783.4	248.4
44	62.1	55.2	97.7	589.2	175.9	135.6	112.4	213.8	522.5	271.8	732.1	249.5
45	37.9	58.2	95.7	635.7	179.0	138.7	127.6	184.6	492.4	266.9	761.5	261.6
46	49.0	58.3	101.8	636.8	242.5	122.6	119.5	205.8	498.5	282.0	771.7	254.6
47	68.4	71.4	83.7	586.5	182.1	136.7	97.4	171.4	452.2	291.1	720.4	210.3
48	54.6	46.2	82.7	581.6	195.2	150.8	158.9	204.7	454.3	267.0	734.6	270.8
49	31.7	78.5	81.7	644.2	221.5	119.6	113.6	190.6	501.8	267.0	769.1	192.8
50	44.8	68.4	100.9	616.1	195.9	133.7	97.4	167.4	503.9	268.1	727.8	261.4
51	54.9	63.4	97.9	593.0	175.3	130.7	112.6	205.8	485.8	263.1	731.0	242.3
52	42.8	80.5	114.0	577.9	206.2	109.6	107.6	195.9	464.7	271.2	805.8	234.9
53	40.8	64.4	79.7	535.7	154.2	110.6	115.6	214.1	421.4	255.1	775.6	228.9
54	45.8	50.3	80.7	524.6	210.1	124.8	105.6	169.8	457.8	265.3	746.5	223.9
55	59.0	46.2	91.9	587.3	180.9	126.8	112.7	218.3	438.7	282.5	675.0	227.9
56	30.7	59.4	86.8	563.2	183.0	130.9	103.6	181.9	475.2	279.5	711.0	221.9
57	53.9	80.6	83.8	545.1	178.9	129.9	94.5	165.8	412.6	236.1	742.4	244.2
58	49.9	45.2	89.9	535.1	189.7	120.8	102.6	162.8	414.7	255.3	659.7	225.0
59	51.9	61.4	95.0	556.4	204.9	140.0	114.8	176.0	438.0	275.6	684.1	237.2
60	53.1	64.5	87.9	546.4	167.5	125.9	106.7	149.7	411.8	271.6	711.5	232.2
61	54.2	51.3	75.8	585.9	190.8	95.6	111.8	193.2	409.3	279.7	642.9	229.2
62	49.1	53.4	85.9	526.4	193.9	102.7	91.6	159.9	459.9	246.4	642.0	211.0
63	61.1	38.2	80.9	585.1	195.9	101.7	121.9	156.9	465.0	234.3	721.0	237.4
64	39.2	33.2	91.0	523.5	190.9	98.7	112.8	148.8	392.7	247.5	638.2	206.1
65	60.5	47.3	75.8	488.2	174.3	80.5	88.6	171.1	403.5	239.5	651.4	227.3
66	50.4	51.4	90.0	516.0	199.9	111.9	64.3	150.9	430.9	280.0	641.4	215.2
67	39.5	49.4	65.8	524.2	189.8	102.8	123.0	201.5	441.7	264.8	636.5	211.2
68	68.8	39.3	74.9	478.8	185.8	85.6	104.8	137.8	405.3	226.4	635.6	188.0
69	51.6	35.2	74.9	483.9	180.7	91.7	97.8	168.2	428.7	221.8	637.7	205.2
70	44.5	62.5	53.6	531.6	171.7	104.9	85.6	157.1	426.7	238.0	620.6	181.0
71	55.7	56.5	70.9	507.4	179.8	105.9	113.0	171.3	398.4	239.1	630.9	205.3
72	56.7	51.4	107.3	483.1	171.7	77.6	100.8	147.0	421.8	210.8	676.5	195.2
73	61.8	47.4	63.8	494.4	175.8	87.7	104.9	184.5	399.6	242.2	613.9	203.3
74	58.7	55.5	87.1	480.3	194.1	107.0	112.0	154.1	368.3	235.2	596.7	227.7
75	58.8	40.3	65.8	435.8	173.8	89.7	84.7	165.3	363.7	258.5	610.0	197.3
76	36.3	58.6	81.1	438.9	183.0	115.1	117.1	168.4	353.6	210.9	604.0	215.6
77	57.4	51.5	70.9	459.2	165.8	94.8	97.9	163.8	364.2	200.8	618.3	206.5
78	32.0	52.5	68.9	440.1	173.9	92.8	102.0	150.6	323.3	207.0	603.2	178.2
79	45.2	44.4	51.7	472.6	168.9	101.0	68.5	141.5	361.9	216.1	549.6	207.6
80	48.5	54.5	75.0	477.7	187.2	82.7	104.0	160.8	340.7	222.2	577.1	214.7

Central Lung In-DTPA Counts (CF IS)

	Patient											
Frame	1.0	2.0	3.0	4.0	5.0	6.0	7.0	8.0	9.0	10.0	11.0	12.0
1	34.2	59.2	67.0	522.7	120.6	104.2	222.2	177.2	338.1	211.6	793.7	316.9
2	31.2	68.2	46.0	535.8	131.6	118.2	222.3	175.3	343.1	198.7	718.0	310.9
3	33.4	39.2	33.0	514.9	109.7	108.3	207.3	173.7	306.4	199.7	714.2	295.0
4	31.4	44.2	51.0	468.9	97.7	120.3	212.3	154.7	303.4	180.7	684.3	291.0
5	23.4	46.2	45.0	445.0	96.7	101.3	221.4	136.7	304.5	200.8	701.4	305.1
6	33.0	47.2	50.1	556.2	93.7	94.3	192.4	119.7	297.1	203.8	646.5	260.1
7	24.6	50.3	62.1	524.2	113.7	87.3	171.4	131.4	275.1	188.8	617.5	249.1
8	25.0	45.3	61.1	469.2	98.7	89.3	183.5	108.3	235.1	198.9	613.6	277.2
9	28.2	44.3	49.1	447.3	120.8	69.3	148.4	131.0	285.2	211.9	608.7	252.2
10	31.7	48.3	50.1	489.4	119.8	63.3	112.4	132.2	255.2	192.9	536.7	253.2
11	29.7	41.3	47.1	410.4	91.8	62.3	132.5	118.2	223.2	184.9	508.0	232.2
12	36.9	49.3	43.1	447.5	110.8	68.3	105.4	115.2	239.3	207.0	518.7	244.3
13	31.9	15.2	55.1	460.6	94.8	63.3	109.4	120.3	234.3	183.0	460.6	205.3
14	34.9	37.3	44.1	457.7	89.8	61.3	114.5	100.4	200.3	175.0	440.7	209.3
15	20.7	38.3	54.1	446.8	124.9	77.4	114.5	95.4	260.1	144.0	444.7	200.3
16	20.7	31.3	41.1	460.9	98.9	50.3	129.6	141.6	250.1	187.1	440.8	185.1
17	18.7	31.3	50.1	460.9	103.9	65.4	119.5	131.0	255.1	188.1	409.8	223.3
18	27.3	41.3	51.2	437.0	125.6	61.4	100.5	129.0	248.2	179.2	412.9	198.2
19	50.0	36.3	54.2	440.2	106.5	66.4	105.5	119.6	248.2	170.2	378.0	197.2
20	25.3	23.3	42.1	448.1	100.5	62.4	103.6	115.6	225.2	148.1	336.0	197.3
21	27.3	33.3	36.1	444.2	104.6	64.4	87.5	99.6	253.3	157.2	396.2	174.2
22	36.3	27.3	50.2	498.5	95.6	44.4	80.5	110.6	259.4	180.3	421.4	183.3
23	26.3	35.3	40.2	403.2	101.2	60.4	82.5	105.8	243.4	193.2	390.3	196.4
24	29.3	41.4	41.2	404.3	116.3	52.4	80.5	102.2	203.2	189.2	398.4	168.3
25	26.9	34.3	44.2	436.5	103.2	54.4	82.6	107.3	241.4	154.1	377.4	180.6
26	25.5	29.3	33.1	469.7	94.2	52.4	88.6	102.3	222.4	177.2	331.3	177.6
27	34.4	33.4	41.2	454.7	105.3	49.4	73.5	101.3	209.4	194.3	358.5	177.6
28	24.3	27.3	40.2	426.6	101.3	47.4	95.7	107.3	277.7	191.3	357.5	170.6
29	25.3	22.3	55.3	455.9	118.4	55.5	85.6	86.2	226.5	148.1	356.6	158.6
30	35.4	20.3	37.2	442.9	90.3	48.4	86.6	95.3	207.5	166.3	324.5	160.6
31	32.4	30.4	39.2	421.8	83.2	42.4	71.6	92.3	222.6	135.1	314.5	167.7
32	28.2	27.4	43.2	431.2	109.4	52.5	57.5	93.3	235.7	142.2	365.8	155.7
33	37.4	26.3	52.3	387.2	97.4	59.5	57.5	93.3	219.6	179.4	331.7	169.8
34	35.4	37.4	31.2	402.3	103.4	44.5	70.6	86.3	198.6	149.3	349.8	133.6
35	22.3	25.4	45.3	433.6	95.4	41.4	57.5	74.2	230.8	161.4	321.7	146.7
36	28.4	26.4	37.2	432.7	92.4	45.5	49.5	94.4	213.7	151.3	333.9	136.6
37	24.4	38.4	35.2	453.9	104.7	42.5	67.6	81.5	207.7	175.7	313.8	153.8
38	37.4	26.4	43.3	419.7	81.5	39.5	73.7	101.7	184.6	168.7	304.8	133.7
39	32.8	24.4	36.2	385.6	88.6	62.6	62.6	103.7	186.6	153.6	341.1	125.6
40	26.8	24.4	23.2	403.8	98.7	37.5	56.6	104.3	211.9	145.6	273.7	139.8

41	24.8	34.4	45.3	421.0	74.5	33.4	45.5	89.2	155.5	143.6	328.1	148.8
42	21.8	37.5	37.3	448.2	88.6	34.4	62.7	84.2	155.5	141.6	329.2	137.8
43	31.8	23.4	20.1	417.1	57.4	51.6	62.7	112.4	166.6	151.7	304.0	124.7
44	31.0	30.4	33.3	402.0	64.5	45.5	37.5	94.3	197.9	135.6	290.0	133.8
45	20.2	25.4	30.2	429.3	90.7	36.5	66.7	94.3	182.8	129.6	319.3	139.9
46	32.3	23.4	27.2	413.3	117.9	24.4	52.6	87.3	191.9	149.8	344.5	127.8
47	34.7	25.4	28.2	371.0	88.7	42.5	43.6	76.2	164.7	138.7	322.4	95.6
48	22.6	22.4	23.2	384.2	98.8	44.6	66.7	72.2	190.0	138.7	303.3	130.9
49	16.5	37.5	27.2	440.7	99.8	24.4	54.7	78.3	186.0	137.8	329.6	107.9
50	21.6	23.4	36.3	399.4	93.8	47.6	39.5	70.2	186.0	138.8	303.4	133.1
51	31.7	19.4	41.4	402.5	76.9	35.5	50.6	75.3	187.0	138.8	326.7	138.2
52	23.6	26.4	35.3	394.5	103.7	38.5	56.7	78.1	167.9	138.8	327.7	118.1
53	19.6	33.5	32.3	372.4	72.3	28.5	44.6	89.2	172.0	141.9	323.7	125.1
54	18.6	32.5	21.2	362.3	87.6	32.5	42.6	82.2	174.0	132.8	315.7	106.0
55	32.7	22.4	17.2	394.7	84.6	37.6	43.6	96.3	179.1	149.0	240.1	112.1
56	18.6	20.4	39.4	376.6	95.7	39.6	49.7	89.3	171.0	144.0	283.5	104.0
57	19.4	34.5	20.2	350.4	90.7	55.7	47.7	73.1	177.1	150.1	289.6	125.2
58	21.4	17.4	29.3	366.6	83.8	53.7	51.7	71.1	172.1	138.0	238.2	113.1
59	27.5	25.5	28.3	357.6	97.0	42.6	49.7	84.3	184.3	141.0	269.5	136.4
60	23.4	29.5	26.3	362.7	74.8	42.6	46.7	58.0	169.1	150.1	239.3	111.1
61	28.9	23.4	21.2	373.9	76.8	44.7	41.6	95.4	157.2	162.3	248.4	114.2
62	28.9	27.5	29.3	354.8	84.9	37.6	49.7	60.0	210.8	121.9	236.3	96.0
63	28.9	25.5	35.4	357.9	98.1	22.4	56.8	67.1	178.5	117.9	296.0	101.1
64	17.6	25.5	19.2	363.0	91.0	13.3	57.8	63.1	179.6	128.0	259.6	97.1
65	28.7	24.5	19.2	330.7	78.9	23.5	37.6	69.2	178.4	110.8	265.8	111.2
66	23.7	34.6	26.3	331.5	99.5	34.6	32.6	71.2	181.4	138.2	241.5	108.2
67	22.9	23.5	29.3	360.9	77.3	32.6	50.8	90.4	177.4	128.1	249.7	98.1
68	22.9	22.5	32.4	298.3	70.2	23.5	54.8	60.1	168.4	104.0	256.8	101.2
69	27.9	27.5	33.4	318.6	81.4	28.5	44.7	68.2	168.4	105.8	268.0	97.1
70	27.9	20.4	20.2	339.9	67.2	18.4	47.8	69.2	170.4	132.2	259.9	83.0
71	20.9	22.5	16.2	299.4	83.4	21.5	52.8	80.4	151.2	134.2	261.0	99.2
72	30.0	28.6	37.5	324.8	87.5	33.6	47.8	69.3	165.4	100.8	274.2	84.0
73	19.8	30.6	18.2	309.7	81.4	35.6	48.8	73.3	166.5	120.1	240.8	107.3
74	24.9	27.5	30.4	307.7	88.5	33.6	43.8	64.2	127.0	132.3	250.0	125.6
75	32.0	15.4	21.3	279.4	90.6	33.6	44.8	77.4	151.7	130.3	252.0	93.2
76	12.4	29.6	15.2	264.2	81.5	24.5	37.7	62.2	130.5	111.0	243.0	105.4
77	29.4	22.5	23.3	295.7	77.4	37.7	43.8	83.9	143.9	106.0	241.0	100.3
78	18.2	27.6	13.2	296.8	76.4	16.4	48.9	54.5	134.8	96.9	272.4	94.3
79	26.4	27.6	15.2	322.1	80.3	29.6	32.6	55.5	156.1	103.0	228.9	103.4
80	21.5	20.5	20.3	321.2	76.2	15.4	43.8	76.8	129.8	119.2	239.1	125.7

Whole Lung Tc-SC Counts (CF HS)

	Patient										
Frame	1	2	3	4	6	7	8	9	10	11	12
1	782.37	544.84	854.95	2490.8	1400.92	1834.29	3649.6	6223.9	1570.9	6946.5	2259.5
2	797.59	582.34	929.15	2407.0	1413.96	1850.51	3534.9	6334.3	1614.5	6957.9	2107.1
3	788.05	465.76	804.04	2431.6	1513.24	1853.91	3459.0	6498.3	1532.3	6781.0	2244.4
4	770.15	470.12	772.21	2379.9	1504.16	1916.02	3394.2	6270.9	1613.5	6790.0	1998.6
5	751.17	428.26	737.62	2373.4	1396.47	1825.31	3121.5	6240.3	1558.8	6440.3	1965.5
6	742.19	480.85	732.70	2393.6	1428.58	1924.18	3016.4	6173.9	1519.3	6258.3	1961.5
7	756.83	463.18	683.96	2320.0	1457.75	1783.50	3111.4	6312.9	1537.3	6433.1	1959.5
8	734.70	454.97	781.51	2252.0	1485.94	1894.73	3105.6	6357.1	1524.8	6348.0	1955.3
9	730.75	414.13	663.61	2442.7	1503.07	1820.67	2751.3	6183.5	1555.6	6365.9	1883.1
10	747.44	421.00	721.88	2273.1	1455.05	1840.75	2828.4	6144.4	1446.1	6280.6	1971.0
11	787.66	402.17	724.42	2293.0	1310.14	1739.05	2719.1	6278.9	1453.0	6008.4	1803.9
12	691.45	424.57	629.18	2325.1	1334.99	1724.86	2743.3	6026.9	1481.7	5481.4	1817.8
13	684.26	410.71	602.00	2275.1	1410.54	1720.80	2670.7	5933.1	1336.7	5655.2	1844.3
14	635.55	322.71	677.52	2151.5	1413.75	1672.22	2678.9	5945.0	1454.4	5341.1	1751.0
15	744.74	362.80	651.53	2219.1	1433.93	1598.08	2670.7	5980.1	1205.9	5084.0	1758.7
16	654.75	404.02	643.54	1967.3	1357.45	1426.18	2619.3	5719.7	1256.8	5009.9	1760.4
17	703.67	408.72	640.90	1717.2	1324.56	1734.63	2577.0	5780.3	1256.6	4758.2	1713.8
18	681.65	355.26	698.29	1732.7	1341.06	1527.12	2471.9	5998.1	1279.4	4700.8	1668.4
19	699.65	327.26	625.42	1651.6	1414.15	1557.34	2541.1	5948.2	1214.2	4507.4	1653.0
20	692.95	309.54	612.80	1563.4	1246.50	1586.77	2541.3	5908.4	1180.4	4211.5	1568.6
21	703.29	368.76	571.10	1524.1	1265.67	1450.01	2540.9	6060.9	1207.0	4051.4	1749.4
22	607.95	340.53	628.16	1357.8	1178.00	1500.49	2516.3	5886.9	1369.9	3975.2	1751.5
23	590.93	345.22	694.07	1308.2	1229.24	1517.61	2365.5	5497.7	1287.2	3979.2	1668.0
24	596.21	387.19	650.28	1346.4	1263.97	1600.17	2482.9	5602.9	1232.4	4103.2	1681.6
25	511.27	378.66	657.81	1314.1	1143.37	1372.13	2369.8	5731.9	1225.7	4104.7	1635.5
26	600.55	359.37	592.82	1202.2	1108.87	1520.99	2404.8	5820.3	1216.0	3974.7	1538.8
27	658.12	357.96	649.45	1242.7	1147.47	1495.51	2458.4	5792.2	1139.4	3797.8	1604.8
28	629.00	292.85	684.45	1210.3	1190.60	1389.75	2251.8	5861.6	1116.0	4103.4	1656.7
29	552.58	416.56	644.85	1199.6	1111.79	1520.37	2250.9	5885.1	1163.9	3874.0	1563.3
30	675.45	377.38	701.82	1226.4	1220.29	1506.02	2285.5	5830.5	1051.8	3908.9	1602.6
31	587.50	274.57	713.35	1106.6	1115.77	1586.09	2247.1	5812.2	1104.3	3799.7	1606.2
32	607.76	312.54	572.98	1196.0	1190.10	1402.66	2271.9	5878.0	1048.9	3772.1	1584.5
33	567.11	243.66	582.70	1142.9	1306.26	1583.41	2083.3	5795.7	1008.8	3758.0	1696.0
34	641.51	288.08	610.67	1105.8	1198.97	1451.23	1996.2	5775.0	973.7	3900.2	1571.4
35	605.87	320.62	637.84	956.9	1178.72	1386.86	2076.9	6056.4	1056.9	3863.7	1567.3
36	565.06	239.39	620.94	1009.6	1090.39	1490.15	2056.2	5979.1	1117.2	3766.4	1570.3
37	581.36	275.46	589.56	1083.5	1180.69	1476.99	2147.3	5736.1	879.8	3846.2	1636.3
38	559.80	322.23	668.20	959.3	1158.54	1452.93	2075.0	5716.3	948.4	3705.2	1613.2
39	560.68	229.47	568.96	1056.8	1088.65	1512.73	2063.5	5538.5	989.8	3780.4	1615.9
40	558.24	266.77	569.20	1020.7	1143.68	1406.84	2104.2	5712.8	951.7	3889.0	1653.7

41	520.22	236.28	647.56	874.1	1097.30	1469.58	2009.4	5610.9	964.1	3919.4	1577.5
42	549.78	261.42	613.58	957.3	1113.53	1484.64	2128.2	5689.0	879.4	3812.1	1466.0
43	561.06	314.95	685.96	965.9	1010.70	1501.40	1905.2	5571.7	902.5	3814.2	1508.3
44	551.73	208.60	639.26	853.7	1110.71	1478.32	2049.5	5514.4	882.0	3688.9	1505.0
45	579.86	272.27	621.06	948.5	1079.22	1415.12	2079.6	5695.2	825.7	3822.7	1524.9
46	583.92	238.84	642.38	922.4	1147.24	1392.59	2073.3	5746.5	966.5	3913.2	1501.9
47	547.58	221.26	571.97	933.3	1067.39	1507.64	2017.1	5593.8	895.2	3716.0	1516.8
48	479.04	207.92	574.03	842.1	1157.10	1461.87	1963.7	5654.1	862.8	3742.8	1557.5
49	602.74	169.39	664.86	923.0	1126.39	1470.27	2074.9	5683.0	900.1	3676.0	1488.6
50	538.45	206.14	500.11	859.8	1203.72	1455.84	1982.4	5498.9	861.8	3698.2	1593.5
51	526.91	232.31	585.39	736.6	1110.99	1455.98	1963.4	5666.3	868.8	3583.7	1357.2
52	492.29	245.92	529.58	898.9	1136.23	1395.03	2045.1	5719.8	860.9	3624.9	1437.1
53	538.76	179.26	505.29	843.2	1098.81	1517.87	2005.7	5732.5	853.7	3510.3	1437.8
54	468.67	255.89	531.14	884.1	1137.81	1599.08	1875.4	5394.0	891.6	3680.1	1500.3
55	542.76	244.69	639.09	811.6	1167.52	1501.20	1951.9	5476.4	821.0	3510.2	1464.7
56	454.26	207.79	702.05	842.4	1168.35	1504.92	1937.6	5460.3	834.0	3652.8	1542.3
57	546.29	194.56	678.21	788.4	1128.64	1550.42	1900.4	5626.9	814.2	3510.1	1503.8
58	467.60	143.87	616.16	814.5	1090.73	1567.47	1968.0	5624.7	864.7	3684.7	1497.1
59	538.55	166.78	593.45	711.7	1096.80	1496.25	1867.5	5626.0	911.8	3706.5	1507.5
60	453.44	162.86	564.59	725.2	1146.01	1524.86	1873.4	5501.2	799.6	3577.2	1504.2
61	491.84	222.01	626.47	702.7	1187.49	1336.93	1876.6	5682.4	870.8	3465.1	1534.9
62	558.24	142.13	633.09	801.3	1053.96	1442.82	1913.0	5595.0	932.5	3450.9	1486.3
63	577.50	166.59	611.88	658.8	1110.53	1429.61	1809.0	5331.4	753.2	3439.9	1460.0
64	454.32	156.15	596.69	686.0	1143.85	1361.23	1784.3	5560.2	845.2	3369.7	1534.8
65	547.53	135.61	501.17	668.0	1156.73	1353.54	1720.8	5571.7	816.1	3404.3	1480.5
66	498.93	196.96	588.67	664.5	1092.12	1412.17	1757.3	5569.4	846.9	3473.4	1482.7
67	495.50	159.07	595.10	647.5	1111.51	1319.62	1786.7	5450.7	850.1	3472.2	1473.2
68	570.22	167.30	595.07	622.0	1012.75	1502.23	1858.0	5473.2	828.2	3532.8	1470.8
69	489.61	207.97	646.19	563.6	1110.15	1401.24	1787.9	5467.0	721.2	3547.6	1459.2
70	539.46	185.89	591.80	636.8	1101.53	1317.54	1686.9	5515.3	863.6	3528.6	1515.0
71	530.69	150.58	579.87	584.3	1116.70	1340.13	1765.5	5580.4	863.7	3473.4	1453.5
72	523.42	170.63	594.62	615.3	1034.26	1443.94	1607.0	5437.3	865.5	3561.9	1492.9
73	494.87	202.51	652.33	595.3	1079.59	1392.50	1824.9	5608.4	853.5	3362.6	1515.1
74	517.41	206.05	693.41	553.3	1013.18	1427.96	1794.2	5644.4	777.3	3428.9	1351.9
75	495.17	217.14	639.62	676.9	1031.41	1336.92	1527.9	5542.5	817.5	3393.5	1458.9
76	528.25	196.53	663.69	655.2	1139.18	1444.97	1580.0	5655.4	832.7	3359.5	1510.6
77	497.16	174.19	677.38	635.9	1030.59	1391.94	1604.0	5576.6	810.5	3296.5	1524.0
78	550.91	145.95	649.15	606.5	1069.57	1362.30	1573.5	5489.0	825.0	3172.8	1490.2
79	417.19	167.37	582.88	548.5	1082.83	1352.86	1598.3	5552.1	830.5	3447.4	1470.0
80	460.24	171.79	542.35	543.4	1007.83	1422.72	1633.2	5621.6	735.6	3427.3	1475.0

Central Lung Tc-SC Counts (CF HS)

	Patient										
Frame	1	2	3	4	6	7	8	9	10	11	12
1	373.79	245.87	355.08	1368.8	607.86	886.29	1773.4	2653.6	766.0	3437.5	1126.5
2	349.11	212.69	377.32	1316.5	608.10	864.90	1760.4	2609.9	734.3	3255.0	1020.6
3	358.83	196.00	298.27	1358.4	735.27	878.49	1623.0	2737.9	669.5	3102.8	1132.6
4	349.40	163.02	307.48	1299.6	695.22	914.05	1638.7	2592.0	739.7	3005.8	991.5
5	360.96	195.56	260.78	1223.9	584.75	868.65	1426.4	2518.0	747.6	2858.7	980.1
6	253.49	198.71	277.43	1293.0	604.30	926.61	1368.2	2669.7	691.2	2684.3	968.8
7	315.54	179.83	263.50	1261.7	657.85	815.30	1401.9	2739.1	710.6	2761.1	915.6
8	304.22	172.01	294.74	1246.4	692.84	860.26	1325.6	2692.3	633.2	2798.3	964.4
9	306.61	156.06	245.27	1362.2	706.19	911.90	1227.3	2665.0	696.9	2661.3	927.1
10	343.55	174.67	258.46	1238.4	593.89	864.54	1186.0	2526.4	621.9	2711.2	924.5
11	315.53	131.40	302.74	1134.2	584.96	750.93	1075.8	2605.8	626.5	2463.8	717.9
12	287.87	110.43	231.55	1158.3	568.38	744.41	1108.2	2481.2	655.0	2247.0	727.6
13	271.14	132.17	218.69	1106.7	507.30	735.85	1081.8	2450.6	597.8	2206.9	848.6
14	274.02	90.61	236.43	1131.8	560.43	745.89	1074.1	2426.3	618.4	2132.8	724.3
15	322.19	121.85	222.16	955.4	564.73	699.44	1071.0	2436.6	519.0	1913.5	701.9
16	290.80	143.73	206.13	924.9	505.33	639.58	1140.7	2265.5	533.4	1837.7	732.4
17	275.87	125.14	220.99	789.6	503.24	735.74	945.5	2252.6	480.0	1771.2	694.3
18	308.21	106.48	247.52	770.9	497.14	633.81	879.8	2298.4	501.3	1769.6	712.1
19	269.39	102.54	273.79	756.7	506.89	734.49	1121.8	2294.9	499.5	1604.5	630.5
20	260.87	82.27	216.53	618.7	374.41	642.04	1056.5	2279.2	467.0	1594.9	635.4
21	273.97	108.37	199.83	602.5	439.86	670.08	1126.9	2474.9	484.4	1502.2	696.8
22	238.07	126.03	271.32	493.3	370.13	695.31	1201.4	2313.1	595.2	1537.6	709.8
23	236.71	98.92	255.12	517.2	424.82	690.02	1084.6	2294.5	567.4	1461.5	698.2
24	247.09	126.43	298.03	566.9	432.11	756.98	1232.7	2239.4	546.0	1526.1	689.3
25	218.23	135.15	224.71	586.9	376.10	640.21	1073.0	2260.6	506.2	1644.9	656.5
26	185.84	98.67	276.05	536.2	345.56	673.75	1115.9	2349.8	540.7	1496.3	642.4
27	222.73	117.77	284.65	520.1	428.82	638.62	1210.3	2314.8	483.5	1452.0	686.2
28	271.10	90.37	219.08	567.6	398.77	615.92	1034.3	2425.5	441.3	1529.3	701.6
29	207.86	116.61	281.28	545.9	418.06	701.43	1041.0	2260.9	441.8	1512.2	652.3
30	259.70	113.15	240.79	602.5	444.00	658.61	1166.9	2220.8	428.7	1517.2	725.7
31	244.33	105.20	316.35	533.9	391.71	700.74	1019.9	2345.1	419.6	1584.9	693.0
32	236.10	104.89	235.46	582.5	405.11	599.15	1106.2	2299.0	392.4	1536.7	670.2
33	235.44	80.04	288.57	524.2	469.71	747.16	920.0	2242.9	367.0	1495.7	673.0
34	228.42	81.49	325.51	522.5	426.98	609.00	924.8	2157.9	366.2	1579.1	684.0
35	230.16	76.86	359.56	432.5	413.02	682.75	939.0	2299.9	401.4	1659.5	658.5
36	211.13	59.31	330.34	468.6	394.96	670.92	893.8	2309.9	400.9	1628.5	669.4
37	245.24	83.03	315.86	428.7	415.08	620.26	865.5	2214.5	309.9	1496.1	701.8
38	217.19	94.46	364.90	507.2	405.49	707.43	944.4	2299.0	325.8	1534.2	701.8
39	210.66	42.97	367.99	501.4	365.64	711.42	847.0	2283.9	339.1	1625.3	727.4
40	200.55	92.34	289.33	531.0	349.07	638.34	878.6	2421.4	306.5	1587.6	802.1

41	204.18	45.82	294.53	434.6	383.70	637.35	733.6	2313.3	342.1	1568.7	712.9
42	191.29	72.76	340.61	474.7	370.11	637.53	880.9	2321.8	314.0	1566.3	644.7
43	213.60	88.57	339.90	497.2	347.78	707.43	795.8	2222.9	323.2	1615.3	663.8
44	200.20	91.25	308.87	432.1	407.10	711.73	813.9	2314.2	288.5	1568.5	683.8
45	221.49	108.25	306.19	483.2	348.35	639.43	899.9	2471.0	272.7	1707.5	737.4
46	208.52	55.32	339.73	418.0	429.97	658.33	902.4	2399.4	297.5	1626.0	696.8
47	256.58	53.24	347.69	441.8	399.59	657.27	730.6	2504.0	288.8	1591.2	708.2
48	189.06	79.80	317.23	388.8	422.44	679.16	724.8	2500.0	258.9	1688.1	749.2
49	198.26	38.31	390.96	435.0	364.93	708.55	808.7	2541.3	311.3	1599.8	697.5
50	201.03	71.30	235.87	423.2	435.96	623.84	857.9	2323.9	278.1	1587.5	764.5
51	199.02	104.03	317.24	359.7	426.15	656.47	823.6	2193.1	267.3	1554.2	678.0
52	213.38	100.34	247.91	417.6	392.68	671.03	852.1	2280.7	276.6	1591.2	711.0
53	189.49	60.49	292.28	439.2	378.80	699.69	823.1	2204.1	223.0	1673.4	709.7
54	164.72	115.09	299.49	396.8	401.37	738.89	690.3	2026.2	280.7	1623.0	781.7
55	178.82	95.85	316.44	398.4	403.49	743.23	622.0	2013.4	257.6	1598.9	765.1
56	171.94	103.25	372.23	389.2	410.94	687.07	648.9	2038.0	269.0	1678.2	774.0
57	210.19	85.16	343.83	395.2	386.08	736.83	711.4	2113.7	223.0	1516.5	742.2
58	171.24	51.02	356.49	384.9	389.88	725.74	880.4	2134.2	291.8	1687.6	780.8
59	201.21	76.77	325.54	337.1	416.71	722.61	859.1	1972.9	277.3	1648.4	754.1
60	148.39	82.28	320.51	317.8	384.19	755.60	770.7	1898.7	245.0	1681.7	720.1
61	189.67	106.82	351.33	302.2	437.07	706.48	875.9	2219.1	253.1	1541.7	765.6
62	203.79	68.25	307.44	353.1	381.95	693.58	826.6	2063.6	291.5	1386.2	719.8
63	199.96	77.11	347.17	323.0	420.40	690.30	799.9	2003.2	244.5	1427.8	769.3
64	182.30	103.17	351.48	323.9	382.28	682.38	760.7	2102.5	255.6	1387.8	786.9
65	199.84	57.91	319.20	259.7	359.78	647.25	793.4	2039.1	234.0	1348.3	749.7
66	239.59	81.80	331.02	270.7	307.49	705.12	785.1	2049.7	255.8	1445.2	765.7
67	162.05	61.00	322.56	255.8	316.19	647.11	756.2	2080.5	271.9	1413.2	722.1
68	189.95	79.54	324.11	209.7	308.57	770.12	809.1	1922.5	270.1	1435.9	723.5
69	183.12	80.79	369.34	269.1	294.17	745.10	722.6	2016.9	214.8	1471.8	709.0
70	196.60	68.76	349.02	244.6	380.29	692.20	706.2	2055.6	258.6	1498.6	712.5
71	237.02	45.98	345.10	221.8	362.79	668.12	797.0	2002.5	304.5	1486.6	679.7
72	218.90	73.56	353.93	239.1	321.06	772.85	656.8	1969.1	306.6	1464.2	787.6
73	194.88	84.31	320.55	177.0	372.73	668.59	769.0	1991.3	292.2	1461.7	716.6
74	199.56	93.68	389.81	230.5	305.52	689.70	699.9	2033.7	265.4	1522.6	697.0
75	187.84	92.46	320.53	251.9	355.14	665.72	594.1	2036.2	245.4	1455.4	794.0
76	207.82	100.38	322.56	230.4	389.10	756.60	588.4	2019.8	263.0	1382.0	773.4
77	170.97	62.08	365.79	247.7	325.86	670.20	570.4	1958.9	297.0	1341.9	773.4
78	177.66	63.06	341.65	231.7	325.89	680.77	515.8	1920.5	245.1	1375.6	754.0
79	207.03	75.11	336.49	240.3	375.31	660.32	531.8	1990.7	282.5	1519.7	737.9
80	230.74	62.18	325.95	199.1	305.60	671.12	712.3	1980.0	221.8	1426.4	743.5

Whole Lung In-DTPA Counts (CF HS)

	Patient										
Frame	1	2	3	4	6	7	8	9	10	11	12
1	133.42	94.02	131.42	366.06	213.04	346.46	633.51	896.15	224.64	1327.83	395.47
2	104.44	119.04	181.46	372.13	203.27	335.52	656.63	953.33	229.68	1224.62	379.53
3	111.46	100.05	178.49	372.19	170.29	294.55	630.73	910.47	255.73	1310.88	384.60
4	134.49	94.07	165.51	336.23	185.33	253.57	617.82	882.61	220.75	1264.07	380.66
5	113.50	94.08	130.71	351.30	161.34	278.64	608.92	829.71	231.80	1193.23	379.73
6	90.49	93.10	155.76	327.34	177.38	291.70	543.96	857.88	243.85	1187.42	403.82
7	123.55	72.09	136.76	356.43	151.38	289.75	545.06	911.10	227.87	1260.72	365.84
8	81.51	78.11	139.79	329.45	176.44	261.76	547.15	816.12	245.94	1129.75	336.86
9	110.57	81.13	133.81	338.52	151.43	264.81	517.20	775.20	246.98	1106.91	352.95
10	131.63	83.14	127.82	324.56	179.51	257.84	468.20	771.32	195.94	1147.17	254.84
11	107.60	62.12	138.86	346.65	150.48	221.82	428.21	786.49	192.96	1136.35	335.03
12	90.59	77.16	120.85	339.70	152.51	246.91	505.44	706.46	189.99	1059.38	331.08
13	98.62	42.09	143.52	329.74	166.17	232.92	466.44	743.66	203.05	1042.53	314.10
14	74.58	66.16	118.69	304.73	150.16	263.03	385.33	755.82	200.08	945.47	281.08
15	117.70	60.16	135.75	312.81	137.15	204.93	426.50	776.00	176.05	826.33	268.09
16	106.69	60.17	129.76	281.77	154.22	210.98	411.53	722.99	206.17	877.61	263.12
17	92.67	60.18	100.69	254.74	138.20	215.03	401.57	739.16	170.10	779.47	268.18
18	94.69	79.25	124.79	229.71	160.30	177.95	375.56	684.11	175.14	766.57	265.22
19	79.66	61.20	95.71	207.68	139.25	185.00	409.74	706.30	187.21	709.51	247.21
20	94.73	45.16	118.81	203.70	140.28	187.04	353.61	670.90	189.25	636.38	250.26
21	61.62	45.16	134.89	195.71	143.32	177.04	381.78	740.27	177.24	589.32	272.38
22	70.67	79.30	119.85	192.73	157.39	175.06	358.75	688.60	167.23	629.58	218.22
23	75.70	64.25	97.79	179.71	120.27	157.02	351.79	723.86	166.26	564.43	216.25
24	84.75	72.30	104.83	172.71	101.22	170.10	349.84	612.52	157.25	549.46	232.36
25	56.64	60.26	108.87	166.71	99.23	146.03	305.71	651.80	156.27	574.66	230.39
26	89.80	57.26	106.88	149.67	114.31	165.14	317.82	619.76	178.40	539.61	242.48
27	71.73	69.32	83.79	160.74	97.25	171.19	343.99	630.92	160.34	536.69	207.36
28	63.71	65.31	98.88	157.76	91.24	151.13	351.09	691.32	142.28	519.70	242.56
29	81.81	59.30	107.94	158.79	96.28	144.12	311.95	631.14	157.38	542.90	232.56
30	77.80	59.31	134.09	151.78	115.39	148.16	318.04	677.49	137.31	544.00	223.55
31	74.80	55.29	84.85	144.77	108.58	172.32	297.98	665.54	135.32	491.81	202.48
32	67.77	50.28	110.00	153.84	107.59	161.28	344.29	650.57	131.32	531.11	207.54
33	77.84	46.26	109.02	144.82	106.60	162.32	301.10	634.59	143.41	536.23	224.67
34	66.79	47.28	82.88	150.88	93.55	137.20	265.95	672.92	132.37	477.99	200.57
35	74.85	56.34	98.99	130.78	94.57	121.13	293.16	614.69	151.51	529.38	242.86
36	71.84	51.32	114.10	141.88	101.63	129.20	249.94	627.87	125.37	474.13	205.67
37	62.80	21.13	97.02	116.74	90.57	134.25	272.13	620.94	147.54	520.50	180.54
38	55.76	50.33	100.05	130.85	106.69	149.37	225.87	590.85	119.38	479.32	234.93
39	64.83	35.24	96.04	88.59	91.61	151.41	282.29	625.18	127.45	461.28	197.72
40	75.92	46.32	65.85	111.77	93.64	142.38	285.36	569.91	118.41	501.64	176.61

41	59.82	22.16	94.06	129.91	76.54	186.71	283.39	593.17	137.57	475.54	201.82
42	67.89	55.40	89.04	124.90	86.62	158.54	264.30	653.70	136.58	476.63	199.84
43	37.68	40.30	104.17	121.90	85.63	142.45	246.21	588.33	126.53	435.41	181.74
44	70.94	33.25	109.63	116.88	84.64	135.42	277.49	587.43	108.42	437.50	225.10
45	64.90	43.33	122.75	104.81	93.72	155.60	229.17	604.66	120.53	496.02	211.03
46	77.01	39.31	109.06	100.79	83.66	136.47	260.45	553.36	124.58	464.86	207.03
47	69.96	48.39	102.02	126.01	72.58	134.48	254.45	572.61	108.47	435.71	177.83
48	88.12	27.22	84.90	120.99	77.64	117.36	278.69	590.86	124.62	455.95	160.72
49	40.74	52.44	120.41	119.00	88.74	125.45	279.75	567.76	99.43	431.82	195.04
50	77.06	34.29	102.28	92.79	76.66	148.67	239.45	534.58	118.62	421.81	188.01
51	57.91	38.34	104.31	114.00	77.68	138.61	242.52	581.07	136.79	459.21	214.27
52	67.00	31.28	87.18	107.96	71.64	129.55	263.75	617.50	92.42	491.57	183.03
53	59.94	43.39	98.29	102.93	68.62	142.69	213.33	613.56	101.52	434.14	178.01
54	67.02	55.51	85.19	101.94	74.69	106.38	239.61	589.45	107.59	417.05	164.92
55	55.93	37.35	98.33	107.01	68.65	112.46	233.60	591.57	108.62	388.86	168.99
56	77.14	39.38	98.34	77.74	80.77	111.47	229.60	553.30	105.61	359.65	158.92
57	53.93	23.23	100.38	76.75	72.71	142.79	230.65	551.38	77.35	427.37	176.12
58	64.04	26.26	85.25	106.05	62.62	117.57	230.69	584.80	102.62	368.86	177.16
59	51.92	38.39	125.67	89.91	81.83	103.44	216.59	484.89	94.55	428.52	152.94
60	54.96	25.26	94.37	91.94	72.75	141.86	188.33	606.22	86.49	373.03	181.26
61	63.06	42.44	81.25	98.02	67.71	111.56	222.72	511.33	100.65	384.21	154.01
62	60.04	42.45	79.24	96.02	60.64	127.75	198.50	553.87	88.54	356.98	200.53
63	79.25	24.26	105.54	83.90	80.87	135.86	218.76	509.49	100.68	376.25	189.44
64	50.96	35.39	99.49	85.94	63.70	131.84	201.61	500.48	110.81	362.16	174.31
65	56.02	10.11	103.55	83.93	59.66	101.53	193.55	516.74	93.64	360.20	146.02
66	38.84	27.31	93.45	77.88	59.67	116.72	179.42	525.93	65.34	341.05	180.44
67	48.96	30.35	91.45	70.81	53.61	109.66	216.88	549.29	92.66	360.33	176.42
68	56.05	24.28	76.29	90.05	92.07	74.26	216.92	519.03	76.49	380.62	143.06
69	69.22	13.16	104.63	97.15	80.95	98.56	200.77	502.93	93.70	391.82	161.30
70	68.22	40.48	82.39	85.02	56.68	95.54	186.63	498.97	112.95	370.63	188.66
71	57.09	35.43	106.69	78.96	77.95	87.46	179.58	513.23	101.84	373.73	161.36
72	43.94	24.30	86.46	71.88	46.57	94.56	188.72	463.70	101.85	353.55	149.24
73	45.97	45.57	81.62	75.95	50.63	86.48	212.04	514.42	88.71	344.50	158.38
74	57.12	28.36	103.91	77.99	75.96	84.47	200.94	500.32	113.03	380.00	146.25
75	45.99	39.51	84.69	90.15	82.05	92.59	136.14	452.80	88.74	330.43	147.29
76	44.98	34.45	78.62	55.72	80.04	112.87	195.94	519.75	86.73	345.69	159.47
77	77.42	18.24	103.97	92.21	53.71	102.75	178.75	483.36	96.87	339.67	158.48
78	50.07	36.49	88.78	60.81	62.84	72.36	213.24	481.41	101.96	373.17	182.84
79	47.03	19.26	74.61	52.71	83.12	107.85	198.07	456.15	81.70	298.22	175.77
80	71.38	31.43	96.92	79.08	49.68	93.68	155.52	479.55	82.73	303.34	143.36

Central Lung In-DTPA Counts (CF HS)

	Patient										
Frame	1	2	3	4	6	7	8	9	10	11	12
1	60.81	42.01	60.61	205.64	92.82	158.23	337.86	388.07	116.82	669.12	222.44
2	44.82	51.02	80.63	223.68	85.03	154.25	357.92	429.15	111.84	584.40	213.47
3	42.82	38.02	75.64	201.70	73.04	140.27	322.97	390.20	132.87	646.53	217.51
4	60.84	40.03	63.64	193.73	79.05	121.28	322.02	397.27	104.87	601.61	200.54
5	44.84	43.04	63.66	220.79	72.06	139.32	323.08	351.30	115.90	589.71	197.57
6	39.84	38.04	63.67	203.81	88.09	130.33	263.07	375.39	120.92	590.81	235.64
7	59.87	25.03	60.67	208.85	66.08	139.37	259.11	433.52	101.92	623.95	184.62
8	38.85	34.05	60.68	213.89	88.12	141.39	270.17	355.49	115.96	545.95	171.64
9	55.89	32.05	45.67	208.92	76.12	123.39	251.19	349.54	106.97	551.05	183.68
10	67.92	37.06	50.69	173.90	74.33	133.43	205.15	344.59	91.96	563.17	143.65
11	54.90	18.03	54.70	185.95	60.31	90.37	167.12	344.65	100.99	545.23	162.71
12	45.90	25.05	40.68	187.99	50.30	106.42	209.23	298.62	95.00	476.18	162.74
13	39.89	12.03	45.10	188.02	68.15	104.43	212.27	307.69	97.02	494.30	132.70
14	18.85	32.08	39.50	163.99	67.16	130.51	171.21	311.75	85.00	410.19	126.70
15	52.94	21.05	44.52	175.05	50.13	87.43	183.27	345.89	95.04	338.07	112.69
16	57.96	14.04	56.56	153.02	58.16	91.45	189.32	301.83	78.01	351.16	121.73
17	47.94	17.05	37.51	147.03	65.19	89.46	129.18	313.92	60.98	330.16	110.72
18	35.91	24.07	54.57	125.99	74.23	72.42	126.19	294.91	67.01	316.18	119.77
19	29.90	19.06	31.50	93.91	55.18	87.49	176.38	304.99	80.06	288.14	95.71
20	32.91	17.06	40.54	76.86	49.17	91.51	151.32	255.68	81.08	273.14	114.79
21	30.91	8.03	46.57	99.96	48.17	63.43	181.45	306.91	79.08	242.07	120.84
22	27.91	16.06	39.55	93.95	62.23	82.51	174.46	326.23	65.05	265.20	89.74
23	30.92	17.07	39.56	73.89	44.17	69.47	149.39	322.27	76.10	189.95	98.79
24	41.97	19.08	47.60	80.93	32.13	83.54	175.52	264.09	68.08	245.21	107.84
25	19.89	11.05	41.58	94.00	27.12	68.49	157.48	260.12	64.07	264.33	97.82
26	40.98	18.08	47.61	91.01	40.18	77.55	169.56	277.24	81.16	253.33	102.86
27	44.00	22.10	51.64	74.95	25.12	58.47	140.45	286.33	70.12	221.22	89.82
28	28.94	18.09	42.61	80.99	22.11	76.57	188.71	311.50	54.06	216.24	126.01
29	37.99	24.12	38.59	74.97	29.14	68.54	147.53	283.41	70.15	238.39	104.92
30	27.94	5.03	53.68	67.95	45.23	63.53	169.67	279.44	56.09	248.48	94.89
31	44.03	20.11	32.57	73.99	30.16	83.64	150.60	255.36	54.09	236.46	97.92
32	32.98	16.09	33.58	97.13	44.24	73.60	157.66	277.52	48.06	232.48	96.93
33	38.02	17.10	58.73	66.98	42.24	79.65	154.67	271.54	49.08	230.50	94.94
34	23.94	15.09	38.22	92.14	27.16	69.61	135.59	277.62	51.10	229.54	104.01
35	25.96	11.07	56.34	75.05	26.16	60.56	136.62	259.56	48.09	238.63	106.04
36	27.97	20.12	61.38	73.05	39.24	54.54	115.51	265.64	45.08	241.69	86.94
37	23.95	1.01	48.31	61.99	33.21	61.59	132.64	257.63	65.21	268.91	101.04
38	33.02	11.07	52.34	69.05	42.28	76.70	103.47	230.50	36.03	194.47	101.06
39	30.00	7.05	50.34	46.91	34.23	61.61	128.66	270.81	44.09	220.67	94.03
40	27.99	7.05	39.27	61.02	34.23	73.71	115.59	262.80	35.04	230.78	84.98

41	19.94	11.08	50.35	79.16	22.16	80.77	107.56	273.92	45.12	222.76	103.12
42	32.03	16.12	44.32	71.11	32.23	65.67	104.55	267.93	38.07	240.93	88.03
43	17.93	15.11	53.39	61.05	24.18	65.68	106.58	264.95	43.12	213.77	93.09
44	24.99	15.11	56.43	68.11	38.29	60.66	105.60	290.19	43.12	235.98	111.24
45	21.97	12.09	51.40	59.06	29.23	70.75	103.60	276.13	36.08	234.00	115.29
46	36.08	10.08	58.86	58.06	24.19	54.63	134.86	256.02	41.12	212.88	103.21
47	29.03	18.15	53.43	70.16	17.14	71.78	107.67	280.26	39.11	197.79	93.15
48	24.00	8.07	31.26	71.18	23.19	53.64	91.55	276.27	42.15	229.08	83.08
49	19.97	13.11	59.90	73.21	27.23	52.64	108.71	262.20	33.08	214.00	94.19
50	35.10	8.07	53.86	44.99	28.24	64.75	122.85	231.99	46.20	220.08	103.28
51	23.00	9.08	60.93	56.09	28.25	59.72	114.80	281.46	54.27	253.41	107.34
52	27.04	15.14	57.92	43.99	19.17	56.70	142.06	289.58	38.14	268.59	97.27
53	22.00	13.12	50.86	62.16	32.29	55.71	112.82	250.27	35.12	229.28	79.12
54	34.12	26.24	49.86	75.30	19.18	42.59	86.60	265.45	38.15	206.10	81.15
55	24.03	17.16	54.92	47.04	35.33	47.65	102.77	240.26	35.13	204.12	83.18
56	33.12	17.16	59.98	38.97	33.32	53.71	95.72	240.30	41.19	188.00	87.24
57	28.07	10.10	63.01	38.98	19.19	67.86	99.77	231.25	41.20	221.36	106.44
58	27.07	5.05	50.91	50.10	28.28	58.78	119.99	256.55	50.30	185.04	86.26
59	22.02	14.14	60.01	49.10	35.36	49.70	105.87	208.10	35.15	209.31	75.16
60	22.03	9.09	41.83	49.10	23.24	59.81	107.91	230.36	37.18	186.11	83.25
61	20.01	11.12	37.79	53.15	30.32	58.81	115.00	205.14	27.08	218.48	78.22
62	28.10	9.10	39.82	56.20	16.17	52.76	109.97	216.29	34.16	172.02	100.47
63	21.03	10.11	60.05	49.13	18.20	53.78	106.95	210.26	36.19	182.16	98.46
64	24.06	16.18	43.88	41.05	25.28	59.85	92.82	216.37	33.16	179.16	83.31
65	15.98	8.09	68.16	43.08	25.28	52.79	96.88	240.67	26.09	186.27	78.27
66	25.08	13.15	43.90	44.10	10.11	47.74	92.85	217.45	28.12	179.22	95.48
67	12.95	19.22	40.87	39.05	13.15	47.75	98.93	224.57	37.23	179.25	93.47
68	25.09	5.06	46.95	43.10	28.33	34.60	105.02	227.65	26.10	194.46	69.20
69	24.08	8.10	61.12	47.16	34.41	51.81	85.81	218.58	32.18	168.18	74.28
70	27.12	1.01	43.93	46.15	15.18	40.69	79.75	221.65	39.27	180.36	99.59
71	25.11	13.16	52.03	28.95	38.47	44.74	83.82	232.82	42.31	207.72	84.42
72	18.02	3.04	58.52	30.98	20.25	39.69	98.01	217.68	33.21	190.54	85.45
73	21.06	14.18	48.20	35.04	16.20	40.71	103.09	202.53	37.26	160.20	77.36
74	23.09	15.19	56.31	35.04	23.29	41.73	115.26	195.47	44.36	190.61	62.19
75	19.04	10.13	47.21	35.05	30.39	38.70	76.78	207.66	26.13	159.24	77.39
76	21.07	9.12	42.15	24.92	25.33	49.85	72.74	232.01	31.21	178.52	91.59
77	34.25	5.07	54.31	42.15	16.21	46.82	69.72	202.67	35.26	186.65	90.59
78	25.14	12.16	54.32	22.91	22.30	23.51	88.99	191.55	38.31	180.61	90.61
79	20.07	8.11	37.10	22.91	29.40	53.93	80.89	175.37	28.18	157.32	98.73
80	32.24	9.12	51.30	46.23	19.26	54.95	75.84	197.70	26.16	154.31	76.44

Whole Lung Tc-SC Counts (Non-CF)

	Patient								
Frame	1	2	3	4	5	6	7	8	9
1	3386.79	2854.53	1765.19	3357.17	2184.33	844.15	3358.78	1865.44	2156.30
2	3344.63	2841.65	1751.43	3321.50	2044.27	748.00	3076.55	1819.69	2092.51
3	3276.08	2742.97	1650.34	3273.79	2070.57	804.48	3256.59	1834.23	1965.87
4	3322.97	2747.76	1709.79	3177.82	2054.18	799.84	3267.22	1757.88	1986.02
5	3287.89	2610.85	1725.06	3195.55	1984.91	758.96	3154.77	1710.26	2021.74
6	3135.90	2608.56	1688.24	3235.62	2028.83	899.20	3053.76	1840.62	2027.15
7	3118.19	2449.78	1682.70	2971.26	1940.75	722.12	3119.39	1718.70	1968.24
8	3197.51	2382.86	1683.50	2898.72	1863.76	796.15	2942.26	1837.44	1952.73
9	3128.15	2250.02	1660.89	2880.97	1973.16	780.20	2959.53	1618.44	1948.27
10	3077.10	2295.34	1719.52	2953.96	1906.19	786.44	3008.19	1755.35	2007.27
11	3132.15	1927.16	1599.11	2638.29	1907.94	748.28	2827.47	1761.17	1900.94
12	3029.28	1923.51	1635.24	2709.87	1930.24	687.83	2630.41	1695.39	2006.20
13	3146.45	1956.16	1697.69	2780.44	1869.53	752.50	2647.62	1813.72	1884.56
14	3183.58	1955.88	1637.26	2811.36	1656.26	796.16	2655.65	1660.62	1919.56
15	3030.86	1927.21	1580.15	2708.21	1836.03	733.71	2514.31	1645.99	1858.93
16	3141.85	1838.94	1616.46	2648.21	1698.08	747.78	2458.72	1698.55	1841.67
17	3111.71	1805.17	1701.31	2551.07	1755.42	779.13	2460.43	1765.38	1763.39
18	3000.17	1818.81	1652.79	2611.10	1661.26	691.54	2391.70	1620.13	1760.75
19	2933.64	1803.21	1711.33	2585.53	1638.02	803.12	2308.46	1733.55	1730.39
20	2976.74	1845.98	1636.10	2558.54	1695.64	747.10	2364.29	1732.07	1704.89
21	2833.18	1906.69	1672.36	2585.05	1625.06	660.48	2419.60	1770.28	1736.88
22	2956.86	1833.87	1624.46	2562.09	1780.67	762.79	2637.12	1760.79	1746.74
23	2806.63	1773.72	1725.63	2472.28	1687.80	742.04	2527.89	1842.81	1654.44
24	2811.44	1778.16	1618.43	2527.31	1687.95	758.03	2486.48	1692.79	1710.73
25	2837.49	1721.60	1645.93	2357.33	1712.73	771.48	2449.38	1664.20	1668.40
26	2795.20	1698.16	1534.33	2339.94	1568.87	666.47	2418.99	1747.37	1676.03
27	2961.92	1715.44	1623.18	2266.54	1641.90	671.41	2488.80	1609.80	1647.37
28	2806.06	1559.16	1604.04	2295.33	1638.44	672.70	2439.13	1705.11	1683.15
29	2755.62	1697.11	1599.88	2392.60	1660.69	765.46	2319.58	1779.20	1613.61
30	2814.21	1697.46	1678.25	2164.86	1603.03	661.74	2404.61	1691.53	1801.06
31	2756.42	1612.17	1561.49	2327.60	1523.89	682.51	2296.44	1680.14	1560.84
32	2796.55	1584.04	1583.94	2212.15	1692.35	670.66	2415.76	1654.78	1711.38
33	2787.76	1527.15	1545.77	2146.67	1656.59	701.49	2391.69	1659.44	1653.99
34	2951.42	1528.77	1599.55	2097.29	1625.57	684.80	2382.25	1586.32	1593.98
35	2816.66	1553.51	1529.39	2171.06	1522.87	738.11	2356.72	1620.04	1602.53
36	2767.29	1598.61	1584.98	2074.52	1647.59	725.24	2269.10	1687.16	1657.79
37	2862.99	1529.69	1680.39	2006.38	1630.86	664.18	2333.62	1693.29	1514.92
38	2740.41	1550.11	1587.06	2054.16	1465.99	666.85	2200.84	1706.10	1669.00
39	2763.15	1525.94	1508.79	1922.01	1455.29	745.68	2342.61	1644.16	1686.40
40	2686.62	1567.47	1543.09	2057.46	1524.27	656.92	2292.68	1597.93	1581.33

41	2792.93	1557.94	1613.62	1912.25	1607.89	639.75	2160.75	1654.75	1518.03
42	2708.64	1567.97	1585.53	1895.82	1470.79	706.23	2418.11	1646.67	1637.80
43	2767.18	1518.99	1586.09	1936.54	1487.44	623.55	2219.41	1642.20	1578.92
44	2595.10	1603.92	1534.60	2004.10	1419.14	664.28	2267.50	1622.32	1661.22
45	2864.95	1528.84	1550.40	1915.05	1456.32	633.61	2298.71	1681.14	1470.90
46	2800.57	1558.22	1515.24	1879.45	1459.02	645.38	2295.27	1711.60	1673.01
47	2525.02	1634.66	1533.46	1970.82	1518.75	726.19	2364.13	1624.24	1553.00
48	2724.03	1573.07	1450.83	1811.83	1514.23	676.83	2343.97	1672.01	1612.68
49	2878.87	1517.94	1436.27	1991.26	1368.91	632.13	2238.85	1602.04	1738.34
50	2794.75	1526.85	1552.29	2032.33	1430.18	678.08	2220.05	1583.59	1561.32
51	2760.08	1538.93	1551.96	2017.24	1426.21	671.38	2261.48	1625.93	1583.91
52	2775.08	1477.65	1563.70	1944.29	1468.99	616.00	2142.03	1525.15	1623.43
53	2785.20	1438.76	1560.30	1917.11	1393.65	661.89	2065.12	1444.54	1545.47
54	2550.96	1516.05	1503.83	1927.57	1405.43	666.70	2253.54	1569.14	1560.53
55	2661.74	1469.25	1504.73	1789.53	1391.82	652.34	2165.93	1661.26	1633.55
56	2688.41	1507.34	1592.45	1843.89	1540.02	525.18	2295.94	1567.27	1570.81
57	2627.58	1531.78	1623.53	1859.66	1418.72	629.19	2158.44	1627.77	1624.31
58	2769.71	1358.60	1512.21	1976.61	1359.36	633.54	2151.69	1644.00	1526.51
59	2677.44	1515.97	1515.10	1781.76	1401.14	609.26	2162.44	1543.38	1505.88
60	2756.34	1359.88	1467.39	1949.64	1317.93	661.88	2278.83	1573.45	1547.23
61	2735.82	1515.78	1494.99	1987.40	1372.46	516.50	2077.16	1601.19	1507.89
62	2732.61	1437.91	1470.05	1828.20	1416.09	697.61	2225.39	1622.76	1575.18
63	2797.76	1462.73	1485.67	1913.07	1425.53	636.31	2168.19	1637.58	1575.06
64	2808.97	1526.81	1478.60	1899.38	1472.70	627.16	2070.76	1565.24	1478.71
65	2803.01	1469.47	1510.96	1919.07	1354.15	653.02	2153.06	1563.23	1471.80
66	2635.06	1487.89	1410.31	1801.87	1314.90	562.76	2217.23	1548.55	1661.87
67	2595.34	1413.92	1357.72	1867.56	1366.79	679.60	2207.35	1604.10	1466.07
68	2851.48	1370.62	1355.24	1850.89	1438.95	631.04	2119.97	1573.27	1552.18
69	2783.61	1376.16	1386.14	1834.49	1348.14	671.00	2209.36	1594.93	1435.02
70	2776.28	1469.14	1388.58	1784.20	1383.42	563.31	2114.01	1486.42	1520.36
71	2438.92	1379.54	1492.29	1739.67	1331.38	570.37	2193.72	1494.45	1485.05
72	2699.10	1368.57	1338.47	1792.90	1452.99	557.78	2127.55	1592.22	1505.98
73	2691.30	1375.44	1482.77	1795.89	1360.24	594.74	2077.69	1527.04	1471.96
74	2672.02	1412.98	1441.83	1701.04	1295.01	553.26	2108.42	1590.21	1513.60
75	2653.85	1448.67	1419.03	1858.18	1445.10	643.61	2180.81	1535.20	1467.19
76	2673.80	1470.46	1366.54	1784.39	1367.00	685.87	2158.95	1514.89	1546.51
77	2436.57	1415.11	1403.27	1903.79	1365.10	618.75	2191.28	1581.10	1505.44
78	2747.76	1399.36	1449.72	1860.14	1321.42	594.26	2041.68	1522.32	1463.14
79	2625.05	1461.40	1452.61	1790.47	1379.06	598.93	2154.90	1397.08	1504.14
80	2745.03	1466.51	1387.29	1683.82	1453.48	618.42	2138.47	1420.43	1505.18

Central Lung Tc-SC Counts (Non-CF)

	Patient								
Frame	1	2	3	4	5	6	7	8	9
1	1587.27	1414.86	780.71	1246.66	993.30	328.25	1646.49	807.82	974.32
2	1628.78	1470.37	780.68	1235.76	872.47	284.31	1453.13	829.27	1018.27
3	1632.80	1405.84	770.73	1193.25	906.93	321.02	1421.81	753.75	895.77
4	1500.17	1422.74	716.94	1131.97	863.50	347.35	1426.51	821.68	913.38
5	1526.23	1375.23	765.20	1159.21	912.42	293.84	1427.30	751.33	1004.86
6	1489.43	1260.04	768.43	1122.06	869.89	378.38	1399.08	840.53	972.96
7	1463.52	1176.45	755.61	1053.88	838.37	296.87	1407.99	756.87	919.39
8	1482.25	1075.17	783.07	1031.10	813.53	323.05	1348.60	806.87	900.66
9	1524.66	1031.52	790.23	957.91	832.79	303.32	1402.27	745.04	890.09
10	1521.89	993.66	740.77	1041.55	855.53	306.91	1379.43	771.23	851.37
11	1445.29	859.84	731.14	965.54	800.20	318.29	1271.61	759.31	846.61
12	1336.83	889.61	718.13	953.60	775.93	277.70	1141.85	764.36	875.01
13	1475.43	889.34	724.94	1025.55	734.85	308.95	1149.57	781.02	839.48
14	1430.29	839.99	689.09	1064.67	698.37	267.47	1064.61	759.12	817.68
15	1389.85	839.38	691.25	912.34	774.67	290.17	1038.24	697.34	855.04
16	1519.66	749.04	735.75	991.73	740.42	284.95	972.61	760.38	834.49
17	1468.17	788.69	736.13	921.67	723.51	286.75	1047.57	809.89	767.14
18	1332.45	728.45	707.11	961.19	725.16	267.49	984.50	750.73	757.91
19	1284.65	778.50	719.41	951.54	686.65	281.17	948.97	770.97	743.35
20	1390.65	709.57	703.27	943.24	689.50	294.68	968.05	772.04	711.57
21	1275.91	762.98	710.76	1027.74	693.21	234.72	993.29	753.78	703.64
22	1371.18	793.45	708.64	1060.54	799.48	257.80	1083.71	843.79	740.14
23	1292.65	709.52	786.15	1004.22	724.17	294.79	950.26	851.09	745.88
24	1250.07	647.05	766.49	1016.80	781.61	285.89	921.71	766.41	734.47
25	1245.51	652.72	773.43	901.81	768.59	320.69	886.85	750.31	725.52
26	1297.57	695.92	650.52	910.72	738.13	278.43	909.44	770.35	709.31
27	1358.52	622.72	761.38	930.60	689.50	233.68	905.73	766.18	657.01
28	1356.43	615.33	697.01	900.26	701.10	280.00	933.01	767.48	666.07
29	1294.68	622.17	696.54	902.77	729.78	280.73	830.93	794.09	608.75
30	1361.08	643.21	775.02	926.70	676.97	228.31	856.61	748.22	736.05
31	1337.20	608.72	714.49	952.56	714.73	264.63	828.45	781.09	661.14
32	1349.56	601.94	670.04	826.15	751.26	278.88	838.63	786.54	690.94
33	1318.02	547.28	643.35	832.22	722.79	234.76	922.04	757.54	701.34
34	1432.60	587.45	716.94	781.61	670.30	253.50	858.96	768.76	670.37
35	1306.56	581.04	680.05	849.83	610.36	285.08	852.44	758.19	696.39
36	1331.97	574.53	686.80	878.34	705.29	259.59	793.41	792.68	651.66
37	1358.07	506.59	756.72	829.11	668.99	231.75	920.34	777.54	562.13
38	1325.31	571.48	733.67	768.08	585.12	244.50	782.43	822.93	664.03
39	1298.43	529.76	642.40	751.13	571.89	288.50	841.23	761.58	708.16
40	1139.81	551.80	650.86	817.36	592.76	234.17	872.03	727.70	677.46

41	1363.52	555.54	696.17	772.83	688.18	246.75	866.84	752.80	627.96
42	1254.75	567.12	697.41	750.06	627.54	260.66	873.79	745.53	703.85
43	1315.72	521.80	670.50	724.44	620.90	225.94	831.12	778.01	681.59
44	1260.35	553.25	692.19	748.67	605.53	234.64	903.66	708.42	694.26
45	1304.31	552.37	702.47	791.76	567.65	239.84	829.62	840.52	621.28
46	1306.48	549.11	705.43	717.45	621.40	237.35	874.45	758.24	706.59
47	1201.28	610.33	636.33	695.19	605.12	242.71	902.77	736.32	677.22
48	1273.69	565.37	669.09	715.61	691.32	223.44	912.18	798.79	660.45
49	1356.50	548.97	582.78	779.54	561.08	235.09	852.79	735.37	718.94
50	1322.87	489.22	691.29	750.62	606.93	235.50	822.47	761.47	665.14
51	1243.00	527.88	665.62	735.28	586.09	252.24	774.41	754.64	667.52
52	1270.41	535.52	697.67	765.16	606.48	174.96	722.66	652.34	684.68
53	1262.60	496.13	665.81	734.00	533.09	199.56	734.33	728.71	667.66
54	1232.79	510.67	640.27	760.73	586.43	250.90	824.97	707.73	621.22
55	1209.31	509.47	673.33	666.53	544.13	214.56	824.49	796.76	697.04
56	1247.94	480.48	689.92	679.19	625.72	182.41	814.33	729.81	639.23
57	1157.12	481.31	735.22	709.68	575.26	233.68	759.71	774.74	687.49
58	1325.59	448.14	672.40	668.29	551.82	224.94	814.80	797.75	627.69
59	1313.40	519.77	665.61	676.24	574.64	199.62	764.03	763.11	620.76
60	1281.81	450.19	680.97	723.61	499.85	246.48	906.68	739.70	666.82
61	1256.65	489.96	679.98	708.23	537.18	190.12	786.51	734.46	674.79
62	1256.42	459.41	598.01	679.55	599.88	240.43	829.81	743.37	676.71
63	1262.70	468.48	643.31	733.78	578.76	210.38	761.96	710.16	637.98
64	1326.31	451.05	655.25	701.84	577.70	219.33	770.92	773.59	689.18
65	1272.50	496.32	653.75	671.19	568.82	189.76	777.58	736.27	666.77
66	1172.24	448.19	617.26	716.63	558.52	203.99	802.85	784.99	734.17
67	1188.97	434.36	630.24	633.93	579.11	207.75	779.00	750.26	607.94
68	1288.25	446.54	597.83	677.78	565.46	178.62	714.23	757.30	694.43
69	1273.31	457.96	617.44	726.41	609.91	230.27	771.25	744.21	666.94
70	1286.08	473.15	575.68	732.97	610.53	158.82	704.07	714.26	668.47
71	1148.15	493.19	682.51	703.44	516.32	181.90	793.29	691.88	684.43
72	1196.10	435.94	587.41	688.77	614.61	195.47	714.71	760.61	564.34
73	1239.90	432.98	656.63	682.84	617.10	191.29	743.58	697.63	615.67
74	1310.70	456.99	593.46	658.35	534.27	145.62	759.93	755.42	659.31
75	1235.72	467.47	617.48	693.54	650.37	214.51	764.00	750.19	652.54
76	1282.88	510.00	547.72	723.83	571.87	228.75	723.27	714.67	632.84
77	1168.20	479.23	578.59	768.55	573.01	216.79	799.67	702.55	606.24
78	1193.05	443.00	643.76	738.21	545.99	175.06	685.88	768.95	584.74
79	1157.90	487.44	652.44	713.38	506.47	211.80	725.48	663.21	642.25
80	1230.62	490.86	601.85	601.85	622.73	198.78	713.84	713.07	614.60

Whole Lung In-DTPA Counts (Non-CF)

	Patient								
Frame	1	2	3	4	5	6	7	8	9
1	830.74	506.49	373.86	854.15	382.47	154.03	680.72	381.87	411.27
2	755.86	464.56	382.93	859.30	383.53	142.05	733.85	369.93	420.34
3	778.20	431.62	380.00	779.40	358.58	145.07	656.94	385.00	415.41
4	728.30	440.70	343.04	772.53	353.64	160.11	649.05	378.06	410.48
5	751.45	403.75	371.12	783.67	361.71	151.13	705.21	382.13	418.56
6	705.53	381.79	352.16	787.81	425.84	123.13	642.26	389.20	395.61
7	762.72	382.86	406.29	764.92	307.77	180.22	673.41	344.21	460.75
8	743.82	366.90	369.31	778.07	358.89	130.18	682.54	378.32	376.72
9	718.91	355.95	395.41	799.24	362.96	167.26	633.58	330.31	403.82
10	691.99	364.03	376.45	734.26	373.04	143.25	603.64	352.41	385.86
11	714.15	329.02	360.48	659.25	348.06	153.29	599.73	353.47	364.89
12	639.12	309.04	368.56	677.40	323.07	139.29	587.81	312.44	360.94
13	692.35	311.09	354.59	637.42	296.06	132.30	537.80	345.57	384.06
14	704.49	311.15	367.68	727.75	270.05	135.33	552.93	305.53	378.11
15	698.60	299.17	314.61	617.59	327.24	135.35	528.96	330.65	390.20
16	668.64	303.23	348.76	632.74	311.25	125.34	539.08	347.76	351.16
17	647.69	265.17	344.81	613.79	308.30	160.47	535.16	309.70	352.23
18	661.84	278.26	358.91	638.97	337.44	140.43	460.02	295.71	362.32
19	659.95	218.11	357.97	614.00	308.41	140.46	507.25	355.96	324.26
20	645.01	283.37	337.96	611.10	298.42	144.50	478.24	314.88	309.26
21	608.99	256.32	312.93	607.19	326.58	153.55	501.41	335.01	335.41
22	630.98	281.46	333.06	623.35	300.53	110.42	544.66	365.18	285.28
23	607.00	255.41	340.14	555.19	277.50	120.48	491.54	273.88	356.61
24	596.66	257.46	341.20	560.31	303.65	140.58	510.70	313.09	303.45
25	572.46	269.56	321.18	583.50	294.66	127.55	522.84	334.23	307.52
26	585.61	226.41	361.41	551.46	335.90	123.55	420.48	310.18	299.54
27	555.57	234.49	345.40	563.61	298.78	141.66	475.80	333.34	333.75
28	523.51	263.67	359.53	585.81	318.93	123.59	422.63	295.22	270.50
29	599.98	219.49	325.42	553.75	309.94	104.52	491.04	309.34	301.70
30	546.81	250.69	311.40	557.87	290.90	132.68	463.99	294.31	312.81
31	517.75	217.56	331.56	525.79	284.91	143.76	436.92	295.37	299.79
32	582.19	212.57	312.51	536.95	277.92	121.67	438.00	338.66	313.92
33	553.13	210.59	332.68	518.94	266.91	135.77	461.21	324.64	344.15
34	565.29	213.65	330.73	518.02	308.20	112.66	419.04	339.78	320.07
35	495.98	209.66	328.77	484.91	295.17	128.77	443.26	302.62	264.79
36	516.18	222.77	323.80	458.83	285.16	133.83	426.23	288.58	279.93
37	553.71	212.75	316.81	486.08	280.18	134.86	398.12	291.65	318.22
38	565.88	194.67	295.73	477.11	306.40	126.83	398.19	290.69	292.10
39	559.94	200.74	316.92	477.19	268.19	110.74	443.56	274.64	261.95
40	526.81	196.75	287.77	457.13	281.33	116.80	415.45	327.04	278.11

41	492.66	181.68	276.74	428.01	254.19	121.86	398.40	280.77	277.15
42	538.07	156.53	301.97	492.54	254.23	137.99	339.04	310.03	242.95
43	555.29	175.69	305.05	423.12	237.15	105.78	408.61	320.16	279.26
44	511.05	180.76	332.30	411.10	255.32	144.09	381.47	280.92	260.16
45	531.29	158.62	307.17	421.25	289.63	116.90	393.63	279.96	281.37
46	517.27	166.71	292.10	476.76	273.55	122.97	386.65	254.81	267.31
47	534.50	176.82	266.95	426.43	264.53	107.87	368.57	281.06	251.22
48	474.10	152.65	319.42	458.77	279.70	106.88	361.57	252.88	269.41
49	522.58	143.60	299.31	412.46	267.65	116.98	392.90	264.01	244.25
50	519.65	164.81	293.31	454.89	239.45	93.80	381.87	253.97	247.32
51	484.43	158.79	293.36	421.68	266.73	104.92	384.96	266.12	267.54
52	453.23	148.72	265.16	422.76	239.53	116.03	365.86	271.21	267.58
53	529.00	155.81	296.49	411.73	268.84	116.05	337.66	293.46	278.73
54	482.66	159.88	272.32	464.29	211.35	102.95	334.69	296.54	252.53
55	457.51	158.90	312.74	437.11	240.66	95.90	352.92	263.28	244.50
56	467.68	189.21	269.38	430.12	241.72	110.05	373.18	290.58	229.40
57	512.19	145.82	265.39	413.03	228.63	95.94	331.84	281.55	239.54
58	466.83	117.57	307.85	418.15	248.87	102.01	328.86	291.69	250.69
59	467.92	149.91	303.87	388.92	203.45	99.00	369.33	260.43	240.63
60	436.68	159.03	290.78	418.29	216.62	131.35	318.87	269.57	242.69
61	452.92	131.77	265.57	391.08	206.55	114.19	363.39	266.58	239.70
62	443.91	140.89	272.69	392.16	245.00	106.13	339.20	248.43	265.01
63	470.27	127.78	270.72	391.21	246.05	118.27	331.17	242.41	236.75
64	481.47	173.30	260.65	412.51	255.19	119.31	339.31	253.57	253.98
65	400.65	137.93	278.90	412.58	223.89	103.15	346.45	261.71	253.01
66	429.04	127.84	293.11	438.95	244.15	90.02	320.21	266.81	240.92
67	460.47	142.03	259.77	353.04	214.86	94.08	336.45	235.50	270.30
68	455.49	140.03	263.87	379.41	233.11	106.23	344.60	269.94	220.77
69	456.58	127.91	294.27	377.45	224.04	63.75	336.57	222.42	232.95
70	434.40	131.98	270.03	419.01	241.29	91.09	320.43	241.69	235.01
71	450.67	125.93	273.11	428.19	254.49	90.09	309.35	225.54	209.74
72	462.89	133.04	234.69	340.18	255.54	91.12	336.74	224.56	213.83
73	420.44	133.06	267.13	354.42	212.04	108.35	305.41	266.12	186.53
74	435.71	96.62	273.25	359.54	247.53	105.33	274.06	240.84	207.83
75	449.96	139.18	274.31	379.87	198.95	105.35	295.38	234.81	210.90
76	400.40	136.17	281.45	349.54	218.23	94.22	315.70	236.87	244.37
77	410.60	86.54	267.32	361.76	207.12	93.23	311.70	209.56	208.95
78	422.83	138.24	246.08	362.83	228.44	85.13	299.59	245.06	237.36
79	415.81	129.14	261.33	360.87	243.69	89.20	269.23	221.79	190.77
80	441.23	114.97	232.98	369.04	187.97	83.14	285.50	242.11	200.94

Central Lung In-DTPA Counts (Non-CF)

	Patient								
Frame	1	2	3	4	5	6	7	8	9
1	392.67	258.64	164.63	326.06	171.43	50.01	347.86	155.23	188.83
2	371.73	219.68	166.66	333.11	173.46	57.02	369.93	183.26	192.87
3	405.81	231.72	176.69	321.17	176.49	52.03	317.96	171.29	197.90
4	348.84	228.76	155.71	292.20	148.50	58.04	307.01	170.32	190.93
5	340.89	202.77	161.74	282.24	172.55	58.05	327.08	172.35	188.96
6	328.94	188.79	159.76	303.31	181.59	57.06	294.10	184.39	161.97
7	364.04	184.82	168.80	288.35	125.55	73.09	325.19	162.40	208.05
8	353.09	176.84	185.86	289.40	160.62	57.08	367.30	156.41	185.05
9	336.12	189.89	168.86	279.43	165.66	78.12	306.27	160.45	179.08
10	338.18	190.93	172.90	279.48	170.69	49.08	309.33	149.46	178.11
11	345.25	167.92	179.94	259.49	145.68	58.11	271.31	166.51	172.13
12	286.19	132.87	166.94	245.51	146.70	57.12	267.35	140.49	156.12
13	316.31	161.96	170.98	235.53	117.66	37.08	273.41	158.55	205.26
14	333.40	159.98	161.99	278.67	113.67	48.12	265.44	124.50	158.18
15	316.41	148.98	144.97	257.66	141.77	61.16	232.40	133.54	181.27
16	300.43	168.06	161.04	250.69	136.78	52.14	281.57	151.62	160.24
17	293.46	137.00	162.07	211.62	143.82	66.19	252.54	142.62	172.30
18	293.51	137.02	146.05	270.84	145.85	46.14	208.44	127.59	169.32
19	291.55	107.95	163.13	233.76	124.81	54.18	235.57	156.71	152.30
20	291.60	141.08	155.13	221.76	137.87	43.15	242.63	118.61	128.24
21	273.59	116.02	154.16	245.89	138.90	61.22	227.62	147.73	160.38
22	292.91	130.09	157.19	276.04	140.93	50.19	230.67	181.89	105.20
23	268.86	121.08	158.22	220.87	136.94	57.23	218.66	130.72	171.48
24	300.04	121.10	151.22	239.99	154.03	55.23	225.73	141.78	140.38
25	266.54	118.11	162.30	237.02	135.98	45.19	231.79	160.89	155.47
26	285.87	112.10	185.43	231.03	150.07	55.25	170.56	124.76	147.46
27	273.87	120.16	179.43	253.17	144.07	67.31	216.80	150.90	150.50
28	266.88	137.26	172.43	242.16	144.09	56.27	201.77	146.91	132.44
29	277.98	118.19	163.41	230.14	133.06	42.21	195.77	141.91	161.60
30	286.07	121.22	162.44	252.30	133.08	54.28	203.85	148.97	154.60
31	248.92	80.03	173.52	222.18	137.13	60.32	171.71	137.93	134.51
32	296.23	87.08	130.31	220.21	153.24	51.28	176.77	165.11	138.56
33	256.05	103.18	154.47	247.40	119.07	41.23	182.83	138.99	132.55
34	283.25	96.16	147.46	221.29	138.21	55.32	163.75	166.17	142.63
35	246.08	83.10	156.54	218.31	141.25	46.28	174.85	164.18	119.52
36	261.21	94.18	144.49	209.29	136.24	54.34	181.92	139.06	142.68
37	273.53	104.26	142.50	229.45	142.30	49.31	170.88	128.01	170.88
38	277.61	78.11	157.63	191.25	156.42	64.42	161.85	131.05	134.68
39	241.41	90.20	138.53	218.46	132.28	40.27	183.02	126.04	118.59
40	247.50	93.24	125.46	213.46	120.22	37.26	193.12	139.15	124.65

41	225.38	91.24	136.56	195.37	131.32	47.33	167.98	132.13	126.69
42	250.60	75.14	131.55	211.52	120.27	47.34	151.89	155.32	108.58
43	256.69	79.18	152.72	176.30	129.35	45.33	152.93	162.40	127.74
44	226.51	80.20	168.87	165.25	118.29	58.44	154.97	124.14	143.88
45	239.04	73.16	145.72	203.57	145.52	48.37	161.04	150.36	132.82
46	257.23	63.10	155.83	204.61	128.41	50.40	172.16	133.25	111.68
47	244.16	89.32	124.60	170.37	141.54	45.37	150.01	135.29	108.67
48	238.16	66.14	168.99	201.66	129.46	47.39	167.17	121.20	117.77
49	241.22	58.09	152.88	181.52	114.36	32.27	169.22	121.22	111.74
50	240.26	64.15	132.74	190.63	131.53	31.27	169.25	123.25	103.69
51	218.10	61.13	150.92	186.63	121.46	43.38	146.08	110.16	125.90
52	208.05	63.16	113.61	178.59	121.48	41.37	165.27	149.53	141.06
53	263.59	48.04	152.99	167.52	124.53	49.45	128.97	138.46	115.85
54	199.04	78.32	122.73	191.77	108.40	42.39	138.08	144.54	104.77
55	215.23	83.38	179.29	187.77	120.53	34.32	152.23	135.47	91.66
56	226.37	82.39	127.82	163.57	122.57	47.45	159.33	163.77	114.90
57	251.65	70.29	132.90	174.70	112.50	33.32	121.99	142.59	103.81
58	233.52	52.12	146.05	166.65	119.59	40.40	134.13	138.57	114.94
59	232.55	68.29	135.97	170.72	105.46	31.32	151.33	116.37	115.97
60	214.40	69.31	143.07	177.82	110.53	39.40	147.31	146.71	111.95
61	227.57	57.20	135.01	156.63	96.41	48.51	159.46	121.47	109.95
62	212.45	68.32	116.84	160.70	127.75	43.46	128.16	118.46	119.06
63	214.51	58.23	120.90	165.79	120.70	51.56	150.42	113.42	113.02
64	217.58	72.39	120.92	175.92	132.85	45.50	132.25	112.43	119.10
65	197.39	50.16	142.18	177.98	121.75	22.25	146.43	125.60	106.99
66	194.39	50.17	125.01	204.30	125.82	38.43	122.18	116.51	107.01
67	213.65	62.31	129.08	158.82	112.69	33.38	128.27	123.62	118.15
68	218.74	66.37	123.03	149.74	117.77	46.54	148.53	149.94	112.10
69	207.65	62.34	138.23	154.83	109.69	37.44	128.31	124.67	115.16
70	204.65	64.37	121.05	187.24	124.89	37.45	135.42	119.63	121.25
71	204.68	62.36	132.20	214.60	135.04	36.44	136.46	116.61	91.92
72	223.95	62.37	103.88	158.96	130.00	38.47	112.18	96.39	102.06
73	200.70	64.40	128.20	154.93	119.90	37.47	131.44	132.86	97.01
74	213.90	39.09	115.05	154.96	132.07	36.46	106.14	107.56	102.09
75	209.89	58.35	132.29	169.17	102.72	32.42	126.42	117.71	104.13
76	192.70	71.53	138.40	154.00	121.98	27.36	138.60	111.65	128.47
77	187.67	42.15	132.34	167.20	116.94	35.47	128.49	106.60	88.97
78	207.97	65.47	131.35	156.08	108.85	32.43	116.35	113.71	115.34
79	190.77	66.50	128.33	163.20	120.02	27.37	114.34	112.72	83.93
80	215.14	56.37	107.06	152.08	101.79	29.40	124.50	145.18	99.15

Whole Lung Tc-SC Counts (Pediatric CF)

	Patient								
Frame	1	2	3	4	5	6	7	8	9
1	553.86	500.80	2004.91	792.53	1935.22	1289.22	1842.58	4178.12	1718.72
2	567.37	365.17	2012.72	848.00	1898.13	1365.29	1783.05	4293.89	1812.26
3	576.24	416.56	2070.02	798.41	1977.45	1201.76	1666.52	4234.77	1781.49
4	600.58	397.29	2145.33	833.01	1922.05	1187.63	1568.68	4048.39	1804.84
5	654.57	415.66	1980.03	824.85	1944.57	1215.45	1574.08	4247.67	1785.15
6	583.76	377.04	2081.33	767.09	2004.19	1084.51	1643.43	4142.73	1741.01
7	603.34	343.43	1930.64	733.72	1877.29	1177.40	1521.41	4040.39	1663.18
8	591.26	447.90	2015.00	709.41	1936.65	1149.09	1509.68	4165.27	1795.28
9	601.20	367.19	1938.44	757.49	1874.17	1174.97	1562.12	3986.76	1752.45
10	544.76	365.06	1984.70	740.47	1882.20	1077.98	1494.92	4042.87	1756.07
11	617.09	349.90	1959.94	791.44	1906.14	1031.47	1486.53	4099.16	1731.30
12	490.28	348.04	1841.26	788.74	1892.09	1094.09	1468.52	4114.25	1708.49
13	515.21	362.11	1859.77	748.46	1827.91	1058.39	1486.38	4136.67	1674.79
14	532.37	404.84	1876.60	776.41	1812.43	1056.08	1317.63	4350.10	1710.95
15	589.62	384.76	1994.76	755.53	1757.16	963.26	1362.00	4246.11	1706.15
16	517.58	351.02	1925.14	695.19	1792.26	1125.79	1320.09	4249.91	1676.65
17	556.79	388.03	1923.89	723.30	1861.27	1001.77	1389.82	4220.90	1725.87
18	575.06	372.80	1873.88	678.77	1897.18	936.22	1402.40	4144.00	1710.40
19	588.07	326.93	1843.24	690.23	1851.83	950.46	1278.01	4247.98	1656.00
20	591.13	316.71	1902.21	683.28	1853.84	968.01	1321.75	4143.26	1716.25
21	488.62	413.37	1857.09	667.48	1907.89	882.42	1172.21	4308.88	1700.07
22	514.90	305.63	1905.14	696.10	1894.86	1022.12	1259.75	4147.62	1643.58
23	528.61	307.26	1777.83	658.96	1890.98	942.69	1173.50	3981.85	1725.10
24	522.10	314.93	1927.86	739.81	1844.73	923.87	1152.40	4228.89	1696.81
25	476.30	327.49	1962.78	761.94	1851.76	889.39	1188.93	4148.06	1722.32
26	541.36	294.61	1958.32	687.26	1785.29	904.56	1264.44	4267.68	1747.75
27	502.15	328.94	1952.64	699.93	1821.18	976.91	1264.25	4358.91	1670.50
28	509.78	348.51	1782.55	631.59	1701.84	863.09	1075.42	4243.16	1664.88
29	513.80	345.04	1749.98	638.51	1797.13	874.40	1214.55	4344.58	1695.42
30	520.53	306.89	1807.73	661.78	1838.99	872.18	1149.10	4268.43	1645.08
31	426.05	327.09	1810.68	694.46	1769.57	861.67	1113.50	4145.83	1643.28
32	546.04	309.86	1825.01	678.37	1780.95	952.00	1159.61	4015.99	1647.83
33	492.78	266.95	1907.60	651.28	1676.00	905.44	1186.79	4078.02	1732.17
34	524.52	297.64	1972.15	689.75	1780.95	931.96	1275.46	4201.08	1615.58
35	511.41	381.35	1928.34	694.92	1742.19	772.42	1213.15	4272.04	1764.15
36	420.44	342.68	1900.67	646.09	1670.32	950.06	1245.77	4211.12	1665.56
37	436.80	301.01	1879.98	674.55	1849.85	835.40	1200.31	4313.32	1674.29
38	425.53	293.00	1863.80	675.64	1783.90	873.92	1174.69	4396.25	1702.42
39	460.78	267.83	1845.11	702.47	1682.07	873.72	1205.47	4220.92	1508.88
40	442.32	325.58	1771.86	701.77	1770.72	795.19	1173.32	4267.77	1676.61

41	459.37	273.29	1754.08	703.52	1609.15	802.29	1088.19	4166.14	1696.41
42	444.27	323.58	1914.50	750.90	1688.88	792.80	1227.01	4166.71	1589.75
43	480.87	349.93	1812.57	650.59	1673.99	886.82	1129.24	4310.99	1660.26
44	458.42	369.90	1765.91	765.09	1756.03	813.35	1146.80	4399.41	1621.87
45	491.10	306.58	1939.71	661.17	1867.84	800.13	1113.84	4431.94	1565.33
46	491.10	317.68	1844.60	666.45	1664.84	764.95	1191.01	4323.72	1671.41
47	421.67	316.95	1791.35	606.83	1730.87	840.43	1187.16	4386.24	1521.57
48	411.19	352.26	1888.76	716.99	1733.48	790.85	1096.37	4354.03	1541.67
49	479.01	338.33	1821.98	756.78	1674.00	749.05	1131.13	4164.46	1629.78
50	434.16	263.76	1803.79	605.33	1690.17	768.33	1166.60	4246.47	1567.98
51	536.50	305.05	1792.73	612.40	1678.54	867.84	1156.53	4308.05	1621.68
52	482.79	358.28	1887.52	660.82	1720.50	785.06	1223.73	4324.82	1537.43
53	504.36	320.63	1867.51	647.88	1753.61	849.27	1189.67	4318.03	1488.63
54	542.94	270.70	1804.14	605.45	1761.07	782.48	1254.79	4290.77	1584.42
55	358.25	251.03	1997.03	645.01	1802.13	821.22	873.99	4235.24	1547.90
56	433.29	273.19	1828.22	687.02	1713.17	847.44	1210.87	4354.96	1576.84
57	450.56	352.64	1925.61	676.36	1747.23	824.57	1156.36	4223.88	1585.01
58	449.34	332.85	1828.32	725.90	1744.74	827.78	1189.66	4302.11	1607.58
59	404.60	336.85	1828.41	718.09	1657.52	765.46	1204.58	4316.06	1529.59
60	518.49	321.74	1924.04	625.50	1681.24	786.85	1149.53	4313.58	1596.04
61	482.36	365.85	1870.91	645.71	1639.69	821.73	1092.35	4541.19	1588.69
62	478.82	299.72	1921.51	660.30	1718.83	756.31	1061.19	4613.07	1643.32
63	557.98	324.56	1896.76	636.79	1704.18	731.27	1110.21	4551.71	1513.24
64	522.41	246.86	1869.72	652.27	1714.92	811.20	1035.44	4254.59	1520.07
65	421.59	388.34	1852.05	690.44	1744.21	792.67	1081.70	4292.56	1583.95
66	414.31	328.13	1839.72	630.79	1706.75	883.18	1074.76	4346.50	1605.73
67	454.35	313.01	1911.72	609.26	1684.89	899.76	1138.20	4384.68	1581.72
68	561.18	288.05	1853.53	673.57	1774.49	811.19	1088.31	4415.39	1518.83
69	414.14	274.04	1913.04	554.50	1708.44	842.91	1073.48	4157.65	1510.38
70	390.70	242.01	1803.22	611.94	1755.82	756.68	1096.18	4328.85	1493.76
71	457.58	312.07	1924.08	648.17	1730.78	750.27	1115.14	4235.15	1489.66
72	536.11	291.75	1768.47	603.89	1677.28	901.57	1211.94	4300.82	1493.09
73	458.40	263.41	1866.22	629.35	1642.32	886.48	884.78	4500.89	1510.62
74	446.69	311.90	1902.90	648.19	1704.12	810.95	1103.10	4439.19	1485.44
75	483.15	277.57	1831.61	555.47	1784.91	745.99	1147.18	4447.55	1591.87
76	456.08	270.63	1911.35	550.06	1763.46	817.73	1120.35	4461.55	1452.03
77	481.66	281.02	1962.88	548.75	1641.37	773.53	1052.86	4518.54	1590.18
78	492.77	278.22	1912.38	568.72	1793.78	773.06	1093.44	4639.75	1388.98
79	443.87	256.00	1889.90	559.85	1722.22	840.02	1124.17	4448.67	1547.68
80	473.22	284.16	1882.21	587.38	1664.15	837.48	985.53	4396.45	1433.74

Central Lung Tc-SC Counts (Pediatric CF)

	Patient								
Frame	1	2	3	4	5	6	7	8	9
1	249.74	205.33	726.39	337.99	919.30	552.42	847.18	1822.34	658.68
2	205.34	164.31	677.04	342.95	832.50	553.39	802.44	1907.24	707.96
3	210.71	176.97	686.95	309.89	940.64	519.11	693.35	1800.60	737.26
4	252.61	174.77	705.15	312.00	906.13	463.71	615.77	1720.11	765.76
5	263.02	138.88	625.11	301.75	874.71	512.54	570.73	1756.15	743.90
6	206.76	161.58	658.07	270.18	899.42	450.99	615.69	1849.87	689.06
7	202.92	130.56	596.13	276.52	898.02	497.09	637.96	1833.30	668.09
8	194.39	178.67	657.67	232.75	900.76	458.97	579.01	1846.44	726.94
9	233.30	163.00	585.00	256.82	843.78	515.68	639.62	1702.60	737.45
10	217.79	133.75	606.86	242.25	821.22	421.24	545.03	1791.93	690.05
11	217.28	146.49	600.52	265.67	804.14	429.11	616.52	1900.69	656.73
12	162.88	144.23	565.63	231.39	882.34	416.45	533.42	1878.91	710.83
13	187.22	127.14	550.27	277.78	794.70	421.96	561.37	1909.47	629.62
14	169.40	168.96	567.04	294.52	764.15	399.35	543.07	2083.61	654.45
15	177.46	157.93	594.27	268.06	737.75	427.51	579.16	2014.08	582.58
16	200.94	139.40	616.48	260.55	786.36	492.90	516.03	2065.90	628.75
17	201.32	159.83	597.08	303.10	797.93	411.09	542.99	1972.98	706.96
18	181.50	160.14	576.18	231.31	828.17	421.42	517.66	1912.44	640.85
19	157.81	137.15	599.64	242.08	868.16	379.71	467.46	2028.56	618.58
20	168.19	106.33	588.05	269.83	850.35	407.18	441.39	1987.31	660.97
21	139.14	131.25	559.57	230.83	844.42	346.86	380.27	2113.82	657.82
22	168.07	97.08	596.10	215.31	863.27	372.97	381.15	1954.16	631.56
23	133.40	127.53	624.83	215.14	839.26	407.31	364.34	1963.53	604.02
24	181.82	108.65	600.02	256.19	823.78	335.93	374.92	1994.34	628.79
25	157.58	116.71	666.25	257.23	790.20	295.71	396.35	1931.99	644.79
26	159.01	124.25	654.32	229.54	779.41	307.33	386.09	1940.14	621.81
27	166.65	130.49	647.66	283.45	814.83	345.47	415.22	2048.21	631.88
28	154.37	117.30	511.47	205.92	698.78	335.11	364.15	1955.19	627.33
29	139.78	112.49	502.34	206.63	786.01	344.68	395.86	2054.68	560.93
30	119.74	151.11	597.52	213.29	871.58	348.03	347.20	2031.40	510.70
31	109.33	98.36	576.15	220.96	816.74	348.46	326.69	2078.65	572.58
32	152.15	87.77	591.31	223.93	776.14	345.34	394.44	2041.82	590.54
33	181.76	83.96	572.71	232.97	771.51	342.74	367.48	2038.06	594.12
34	139.50	84.59	639.14	234.91	789.32	391.34	390.68	2136.94	629.13
35	162.59	128.07	684.20	240.36	782.83	275.95	374.19	2078.36	605.98
36	159.88	138.75	535.83	225.05	746.13	395.86	381.57	2023.91	468.47
37	148.89	137.43	555.62	203.14	876.42	302.25	395.74	1994.98	569.70
38	149.09	75.79	617.72	221.85	840.87	297.39	380.60	2074.87	556.30
39	154.25	87.08	491.14	271.00	742.90	330.80	422.55	1913.63	450.50
40	170.78	146.01	586.95	215.71	745.96	296.00	394.22	1998.80	500.22

41	128.51	122.30	516.50	264.79	718.38	253.72	370.89	2066.29	486.84
42	112.42	135.46	581.33	241.41	751.99	278.61	387.90	1987.88	467.18
43	161.12	124.90	584.34	214.02	748.71	300.44	438.78	2013.04	518.41
44	155.19	128.08	617.41	268.90	766.07	281.52	412.15	2167.90	505.57
45	151.93	114.15	626.75	221.51	763.46	312.77	375.60	2220.85	475.97
46	182.18	108.72	608.05	246.20	700.02	268.12	400.34	2240.87	554.37
47	127.11	93.61	596.55	216.54	734.95	273.01	393.14	2180.04	518.47
48	131.98	129.11	561.52	248.74	808.63	264.52	339.11	2076.88	468.28
49	151.71	90.74	546.73	281.53	722.83	250.47	391.59	2053.12	524.04
50	141.24	113.73	545.05	219.19	699.85	297.73	361.69	2110.35	553.70
51	179.57	94.26	612.51	216.41	684.74	338.52	437.06	2111.41	529.31
52	160.65	150.16	589.47	246.01	696.19	321.98	392.65	2218.78	471.63
53	154.80	110.65	543.21	207.53	719.41	317.07	415.25	2170.94	500.07
54	150.86	105.65	573.90	232.65	726.33	304.12	415.23	2136.91	475.08
55	156.86	120.51	647.16	246.43	712.66	289.15	304.91	2116.10	496.47
56	120.79	115.29	581.71	201.86	700.96	306.65	345.51	2164.29	494.42
57	154.36	146.51	604.16	220.03	762.85	255.07	390.11	2148.83	547.40
58	141.39	130.86	590.55	242.19	701.59	270.06	424.14	2209.88	483.44
59	136.13	120.50	669.50	240.42	700.27	295.04	382.18	2163.20	531.18
60	168.76	126.88	712.29	227.42	711.21	303.37	389.94	2204.15	502.03
61	151.89	136.90	756.01	248.37	686.03	305.71	350.07	2395.77	565.63
62	118.22	107.64	664.60	209.69	706.98	246.17	368.89	2383.85	576.84
63	171.08	75.81	693.98	203.32	670.34	189.74	401.68	2367.23	520.76
64	181.04	90.15	731.68	210.74	694.13	262.64	300.59	2152.50	472.95
65	118.36	143.56	623.33	216.78	760.26	239.16	352.53	2252.28	570.02
66	140.39	142.43	598.13	206.15	684.02	300.25	395.12	2240.03	491.10
67	136.36	129.02	567.02	192.36	693.92	301.03	418.90	2291.33	515.71
68	192.18	109.61	674.23	217.39	689.51	233.95	396.72	2223.26	470.00
69	123.33	89.51	679.24	195.28	662.21	249.83	354.58	2192.97	494.31
70	157.15	125.07	655.09	214.11	677.41	230.57	367.67	2245.64	473.97
71	161.14	131.52	726.79	198.21	709.97	254.38	369.87	2196.38	453.42
72	226.22	128.93	726.37	181.97	693.48	298.23	388.09	2042.49	460.16
73	117.38	111.42	725.78	171.67	701.70	285.34	290.38	2459.23	459.45
74	126.06	109.88	798.76	219.60	687.72	244.92	322.64	2469.45	462.68
75	133.22	138.05	678.14	195.95	741.03	226.67	357.63	2384.52	527.26
76	135.31	119.69	671.60	189.08	711.87	273.87	290.71	2366.67	471.60
77	147.61	103.10	598.04	209.00	711.82	189.95	342.16	2394.86	457.49
78	127.67	110.01	600.44	216.44	724.14	209.15	297.84	2532.64	449.59
79	64.69	88.65	590.42	180.89	704.59	240.04	378.36	2381.99	487.41
80	163.85	107.02	583.14	228.62	637.23	259.45	296.07	2364.15	427.49

Whole Lung In-DTPA Counts (Pediatric CF)

	Patient								
Frame	1	2	3	4	5	6	7	8	9
1	124.62	88.02	240.44	122.42	237.04	206.04	286.45	382.27	236.44
2	124.64	62.02	260.29	99.23	238.08	183.06	316.51	374.33	261.69
3	113.66	95.45	265.34	96.25	266.74	223.12	299.55	406.41	254.73
4	80.66	57.44	286.60	96.67	238.76	181.93	280.59	351.44	250.77
5	98.88	55.45	275.64	71.66	245.81	194.97	283.24	427.37	253.02
6	99.70	69.47	255.66	86.69	226.83	195.00	268.28	374.39	249.06
7	88.71	73.49	261.71	80.70	240.89	197.44	260.31	386.87	204.05
8	100.74	66.49	241.73	73.70	206.88	175.44	279.38	389.13	244.14
9	92.94	50.48	239.17	95.75	227.95	184.49	302.47	361.16	213.13
10	102.98	67.52	244.22	60.70	218.98	170.49	265.86	387.27	224.39
11	82.96	69.53	221.22	72.74	208.99	180.74	266.30	393.34	209.40
12	88.78	59.52	232.28	64.73	233.08	150.71	281.18	371.37	228.07
13	87.80	67.35	239.33	66.95	216.08	171.38	244.15	357.40	194.03
14	83.80	50.32	236.37	67.96	230.15	143.55	213.51	341.02	220.13
15	49.33	61.36	242.42	65.97	237.21	139.56	229.19	388.20	230.19
16	66.38	56.35	243.47	74.00	241.26	137.58	188.12	359.19	233.24
17	70.41	57.37	249.53	69.00	190.15	157.66	193.97	338.19	231.27
18	90.48	53.36	221.48	54.97	203.23	155.68	234.12	345.27	209.25
19	77.45	46.95	214.50	69.03	210.09	142.67	218.91	356.36	201.26
20	69.44	50.97	218.55	74.05	235.21	132.66	215.94	327.32	191.26
21	82.50	62.02	212.57	63.03	235.25	133.68	200.92	368.53	216.38
22	82.51	49.99	208.59	72.07	214.21	116.64	198.35	348.52	219.43
23	72.49	66.06	192.56	50.00	221.88	119.67	220.47	350.58	181.32
24	60.25	52.82	229.95	56.43	214.28	96.60	222.52	362.69	199.42
25	72.31	64.88	187.81	55.44	201.87	129.76	197.25	328.61	204.48
26	73.33	49.82	204.91	48.42	205.92	131.79	166.14	343.73	192.46
27	54.25	45.81	226.05	53.45	222.03	108.70	186.26	339.77	189.28
28	51.05	56.87	196.34	42.40	189.91	123.79	194.33	351.89	214.03
29	67.13	70.35	213.46	47.44	204.42	100.70	196.98	341.90	233.16
30	64.13	67.35	207.07	42.02	219.93	96.09	178.92	361.06	210.08
31	72.59	36.39	196.04	47.85	197.85	112.20	163.87	326.94	210.12
32	64.15	50.07	204.12	58.72	160.68	101.15	149.82	316.94	212.57
33	47.07	36.00	188.67	56.72	202.95	110.22	164.33	301.71	189.47
34	36.01	39.83	209.82	49.69	174.22	118.29	201.78	325.70	199.56
35	62.17	53.72	195.37	41.65	192.35	110.26	182.09	317.71	171.43
36	57.15	37.63	184.14	52.52	185.34	105.25	186.15	331.85	201.64
37	57.36	48.11	183.16	36.43	192.42	90.17	197.25	372.16	203.29
38	54.35	56.37	196.08	31.40	188.43	107.10	173.13	325.92	183.19
39	48.32	44.50	181.41	39.46	202.55	91.01	176.18	314.90	188.26
40	49.34	43.50	206.01	47.53	196.55	105.12	158.08	354.03	176.21

41	54.18	53.58	181.88	36.46	205.64	92.05	152.07	278.56	182.28
42	47.14	49.36	194.00	45.73	219.78	91.06	179.29	306.81	177.28
43	66.69	40.30	185.97	41.10	177.71	95.10	151.11	324.99	179.32
44	69.73	40.30	184.59	25.79	194.67	105.19	155.77	316.99	173.51
45	51.60	36.28	204.78	28.82	192.89	85.06	163.66	282.78	149.75
46	55.64	40.52	173.57	46.97	146.55	82.05	159.46	344.31	181.03
47	57.66	66.13	206.86	36.90	185.90	89.12	133.88	302.03	162.91
48	60.70	45.98	162.33	27.83	170.80	73.00	142.57	279.90	166.97
49	55.67	54.26	204.72	36.51	195.04	96.21	132.51	293.06	155.91
50	53.66	31.07	188.01	50.43	177.52	73.03	140.00	336.48	142.82
51	34.50	58.11	172.71	31.07	185.62	95.23	118.03	300.22	150.92
52	62.76	58.12	174.76	41.17	189.69	109.37	151.55	315.41	143.88
53	61.76	38.35	207.89	41.17	165.50	72.05	163.28	291.24	148.95
54	60.76	53.49	150.39	36.74	154.43	78.12	144.33	300.58	138.88
55	42.80	41.59	150.42	36.75	165.56	85.20	121.34	274.58	162.13
56	59.97	53.11	162.56	35.74	189.01	74.11	144.99	297.85	151.05
57	51.91	46.05	183.79	28.07	188.84	88.26	137.95	289.83	149.05
58	45.65	57.98	187.86	43.63	203.01	84.64	157.56	322.20	160.19
59	50.51	56.97	191.94	35.56	161.63	68.49	129.71	276.79	143.04
60	47.08	50.92	161.66	26.27	179.04	73.55	113.57	258.65	158.22
61	49.52	40.83	178.87	20.21	158.25	81.85	124.09	306.19	117.83
62	47.30	46.90	153.83	31.33	149.38	60.85	118.05	320.40	134.02
63	56.41	49.94	168.01	22.24	155.68	77.03	112.01	263.84	110.79
64	46.51	50.15	177.14	34.38	155.70	69.97	110.21	260.85	153.28
65	39.44	41.26	180.20	43.69	150.67	93.24	138.54	274.05	154.31
66	48.75	26.70	186.30	28.52	153.73	77.07	100.33	278.74	144.23
67	60.90	31.36	163.67	37.63	157.81	61.91	91.45	306.11	126.04
68	32.38	37.44	142.66	37.64	142.86	73.05	109.07	289.97	126.07
69	34.61	48.98	161.91	22.46	171.22	55.86	98.36	285.16	140.25
70	49.59	35.42	157.89	36.44	132.79	67.00	97.57	272.26	132.18
71	41.50	41.10	149.41	27.13	155.08	70.05	87.87	252.26	138.28
72	36.45	44.14	162.40	21.06	156.12	54.88	112.18	277.01	163.61
73	34.43	41.11	132.25	29.16	181.87	71.09	77.97	270.17	121.11
74	31.60	35.04	163.67	20.26	149.49	60.97	81.23	263.13	159.62
75	37.48	27.96	183.96	26.95	125.20	54.90	77.39	292.95	119.13
76	21.68	37.89	169.80	26.95	164.74	67.07	97.87	252.48	132.32
77	40.94	32.22	166.79	27.97	138.42	77.22	88.77	260.63	119.17
78	42.77	27.57	149.59	31.42	153.65	64.05	89.80	269.79	128.31
79	43.18	38.32	169.89	18.04	139.48	67.11	84.34	251.59	111.10
80	35.08	36.30	160.80	23.72	147.62	67.12	98.75	259.75	113.15

Central Lung In-DTPA Counts (Pediatric CF)

	Patient								
Frame	1	2	3	4	5	6	7	8	9
1	46.21	46.61	107.42	53.41	116.62	113.42	144.62	192.43	107.82
2	47.22	30.61	91.43	43.41	114.64	80.43	157.65	181.46	116.84
3	38.62	47.62	109.46	42.42	151.28	116.46	140.67	174.49	106.86
4	39.63	26.62	91.46	34.82	129.29	96.47	137.69	163.51	105.87
5	41.04	32.63	89.48	38.83	135.12	87.48	131.11	199.57	93.68
6	37.24	30.63	80.48	42.84	109.11	93.50	127.13	173.58	108.71
7	33.24	30.64	86.50	28.83	123.15	90.91	111.13	196.24	91.71
8	37.25	27.64	88.52	30.84	105.14	75.90	131.18	194.27	96.73
9	39.26	15.62	78.72	41.86	108.17	77.92	136.21	184.28	84.73
10	37.26	20.64	90.76	32.86	94.56	81.94	132.03	195.34	99.57
11	36.27	32.66	80.75	30.86	99.59	91.17	128.84	195.57	104.60
12	32.07	31.67	81.77	32.87	125.66	71.15	116.84	205.62	98.40
13	29.06	34.08	83.79	28.26	87.60	88.80	120.87	189.62	76.37
14	28.07	19.05	79.79	33.28	104.65	71.37	101.84	181.64	83.40
15	16.04	34.09	84.82	33.29	109.68	77.40	109.08	206.73	111.89
16	26.07	20.06	68.79	34.29	98.67	74.40	88.04	189.72	97.87
17	24.07	24.07	77.83	32.29	84.65	71.41	89.46	170.70	95.88
18	38.12	26.08	86.87	21.27	86.67	63.40	102.52	182.76	93.89
19	16.05	14.65	73.84	31.30	95.71	65.41	72.64	181.79	88.89
20	24.08	20.67	85.89	33.31	118.81	49.37	94.73	175.80	77.87
21	29.10	35.73	68.85	25.29	120.84	54.40	77.68	199.92	101.37
22	29.11	15.66	66.85	28.51	96.77	61.43	78.30	185.90	108.21
23	22.09	24.70	59.84	24.50	105.82	47.39	89.35	189.95	58.23
24	14.26	23.70	98.41	26.71	114.87	44.38	96.40	185.97	76.31
25	26.31	27.72	66.28	26.71	104.45	41.38	77.53	181.98	85.37
26	30.34	28.73	68.30	15.67	94.42	54.44	65.49	184.02	71.32
27	16.28	12.66	61.28	22.71	96.65	50.43	87.61	186.06	72.33
28	22.71	21.70	57.28	16.68	79.58	60.49	71.14	187.10	83.20
29	10.65	29.55	75.37	20.70	95.68	49.45	96.08	201.20	89.24
30	24.73	30.76	74.78	21.11	123.84	50.46	69.96	196.21	76.19
31	19.91	17.49	65.75	19.30	84.65	55.49	68.97	180.16	78.22
32	21.32	23.93	70.79	21.12	83.66	40.42	69.98	191.25	80.44
33	22.53	14.68	57.32	22.13	94.74	53.70	72.21	177.40	79.45
34	9.46	16.70	82.48	18.11	96.36	62.77	84.89	184.88	61.36
35	17.30	23.14	69.01	14.69	103.42	48.69	74.85	183.50	59.36
36	18.31	15.09	68.62	16.50	91.36	44.68	73.86	177.50	79.49
37	25.76	7.25	62.60	8.45	99.43	38.65	83.93	194.63	73.06
38	16.71	25.97	63.41	9.46	97.43	41.07	69.85	174.54	66.03
39	9.66	17.52	52.75	12.48	86.18	51.14	62.82	151.41	65.03
40	16.71	15.51	72.50	15.51	82.16	47.12	53.77	192.32	63.03

41	14.70	19.94	56.40	10.47	100.30	39.07	69.89	160.12	51.96
42	17.73	24.17	71.51	15.51	95.29	37.07	75.95	173.25	71.11
43	18.54	13.10	62.46	10.88	87.85	36.07	59.84	176.30	64.07
44	20.55	17.13	69.32	7.86	78.19	43.12	58.64	192.45	63.48
45	19.55	14.11	66.31	7.86	81.02	36.08	59.66	145.12	63.69
46	20.56	17.13	70.35	18.95	63.90	38.10	59.87	190.50	55.64
47	17.54	32.66	67.75	13.31	85.08	47.18	58.87	172.39	72.79
48	21.58	24.60	53.64	6.25	94.58	30.05	44.36	166.37	55.66
49	25.61	18.76	72.81	18.96	83.50	38.12	68.98	169.42	52.64
50	24.61	9.68	65.76	21.38	82.71	38.13	61.53	187.61	68.79
51	16.54	24.41	57.30	12.31	77.68	44.19	52.86	184.61	57.70
52	25.63	24.42	69.42	14.33	70.63	41.17	58.52	193.72	52.67
53	16.55	12.92	70.64	19.58	86.38	37.14	57.93	181.24	57.72
54	15.54	31.09	49.46	19.18	70.25	37.14	69.85	159.07	47.64
55	16.76	16.56	52.49	8.08	78.34	38.16	48.46	168.99	59.76
56	28.07	19.18	53.51	15.75	73.50	21.00	57.96	176.09	53.71
57	23.02	18.38	66.65	10.70	74.73	31.10	41.81	160.97	59.78
58	19.80	24.24	63.83	13.94	87.87	32.32	67.27	177.16	63.83
59	18.79	29.30	62.83	7.88	77.78	39.60	58.79	153.95	47.68
60	10.71	21.22	62.84	5.66	77.80	26.47	52.74	153.98	64.87
61	8.89	14.15	65.89	8.69	71.55	28.09	44.67	188.36	48.71
62	9.10	19.20	61.86	6.67	68.12	30.32	46.49	184.35	43.66
63	20.22	14.15	67.93	8.69	77.64	25.27	57.82	167.20	41.65
64	27.30	18.00	61.47	19.82	70.77	32.35	42.46	162.78	52.78
65	20.22	14.36	72.61	7.69	77.87	33.37	61.69	150.67	48.74
66	24.48	12.95	55.43	7.69	57.65	36.41	41.67	170.12	50.77
67	23.47	12.95	63.33	12.75	77.89	19.22	46.53	167.11	47.75
68	16.19	12.95	49.98	16.80	61.31	30.35	44.32	162.08	32.58
69	13.76	17.61	68.20	10.73	81.56	22.26	31.37	177.90	43.72
70	23.89	13.16	64.17	11.94	48.18	23.28	45.14	171.05	52.63
71	15.79	19.64	55.88	11.54	66.41	36.44	33.81	163.79	52.64
72	13.77	20.65	77.35	9.52	77.55	23.29	42.93	154.91	59.73
73	9.52	12.56	52.05	16.61	89.52	27.34	33.42	166.07	38.48
74	8.51	13.57	60.16	8.30	52.06	35.45	30.38	168.13	69.88
75	11.75	13.57	65.64	12.56	64.22	24.31	29.38	177.07	36.47
76	9.93	16.01	61.60	7.50	71.33	26.34	45.19	140.63	40.53
77	10.94	22.09	55.53	11.75	62.22	30.40	37.09	161.93	39.52
78	21.49	9.32	47.43	6.89	77.43	22.30	39.12	186.28	53.72
79	6.89	20.48	54.54	4.26	56.16	23.31	24.53	150.83	35.48
80	11.56	14.19	49.48	11.15	69.35	37.51	38.32	166.07	42.58
