# Supplementary material for: A Physiologically-Motivated Compartment-Based Model of the Effect of Inhaled Hypertonic Saline on Mucociliary Clearance and Liquid Transport in Cystic Fibrosis
Source: PLoS One. 2014 Nov 10;9(11):e111972. doi: 10.1371/journal.pone.0111972 (PMC4226497; doi:10.1371/journal.pone.0111972)
Supplement: Table S2 — FFCA in CF HBE cells is shown at baseline compared to a 5 ul addition of either isotonic or hypotonic saline. (PDF) [file pone.0111972.s004.pdf]

Baseline		After First 10ul Addition		After Second 10ul Addition	
Filter Number	FFCA	Filter Number	FFCA	Filter Number	FFCA
1	1.13	1	4.253	1	7.885
2	3.117	2	27.988	2	21.222
3	50.664	3	98.877	3	88.943
4	22.817	4	77.022	4	72.003
5	32.405	5	50.968	5	66.111
6	31.776	6	71.512	6	58.667
7	1.668	7	4.358	7	9.212
8	10.115	8	21.571	8	21.77
9	41.487	9	88.91	9	62.135
10	26.114	10	86.051	10	64.341
11	9.762	11	60.04	11	42.305
12	13.921	12	79.626	12	71.721
