# Supplementary material for: A Physiologically-Motivated Compartment-Based Model of the Effect of Inhaled Hypertonic Saline on Mucociliary Clearance and Liquid Transport in Cystic Fibrosis
Source: PLoS One. 2014 Nov 10;9(11):e111972. doi: 10.1371/journal.pone.0111972 (PMC4226497; doi:10.1371/journal.pone.0111972)
Supplement: Table S3 — FFCA in CF HBE cells is shown at baseline, after one 10 ul addition of basolateral DMEM, and after a second, sequential addition of basolateral DMEM. (PDF) [file pone.0111972.s005.pdf]

Isotonic Saline Group			Hypertonic Saline Group		
Filter Number	Baseline FFCA	FFCA After IS	Filter Number	Baseline FFCA	FFCA After HS
1	43.18	51.633	7	41.286	55.689
2	40.381	47.851	8	33.398	58.131
3	39.138	39.228	9	32.672	56.552
4	39.017	53.317	10	31.637	48.474
5	35.673	51.96	11	35.649	54.433
6	37.548	47.271	12	31.535	48.258
13	1.909	4.377	19	3.594	13.669
14	0.797	3.419	20	1.05	9.973
15	7.444	8.873	21	1.813	8.286
16	1.786	9.343	22	3.498	7.901
17	0.603	9.295	23	1.314	7.129
18	0.451	3.549	24	1.447	6.254
25	36.196	13.856	31	32.753	51.759
26	33.154	31.062	32	51.29	54.863
27	35.36	35.569	33	29.524	48.387
28	34.054	38.593	34	20.484	25.678
29	31.39	33.417	35	40.202	43.58
30	32.619	40.89	36	37.282	49.255
